# Exploiting Device Deformability for Fluid and Particle Manipulation

**DOI:** 10.1002/smll.202513205

**Published:** 2026-05-24

**Authors:** Zhiyang Hong, Xiaoyue Kang, Dan Yuan, Nam‐Trung Nguyen, Jun Zhang

**Affiliations:** ^1^ Queensland Quantum and Advanced Technologies Research Institute Griffith University Brisbane Queensland Australia; ^2^ School of Engineering and Built Environment Griffith University Nathan Queensland Australia; ^3^ School of Mechanical and Mining Engineering University of Queensland Brisbane Queensland Australia

**Keywords:** flexible electronics, flexible microdevices, fluid and particle manipulation, microfluidics, soft robotics

## Abstract

The ability to precisely manipulate fluids and particles underpins a wide range of scientific and engineering disciplines. Conventional devices for fluid and particle manipulation are predominantly developed based on rigid materials and platforms due to their high mechanical strength, dimensional stability, and compatibility with established manufacturing processes. However, the intrinsic rigidity of devices limits their mechanical flexibility and adaptability, rendering them unsuitable for applications requiring conformal contact, dimensional control, or dynamic interaction with soft or irregular environments. Recently, emerging soft materials and deformable architectures offer entirely new modes of actuation and control. Despite rapid progress, the field lacks a unified framework that links material deformability with specific operational mechanisms for fluid and particle manipulation. This review aims to provide a mechanistic understanding of how device deformability can be intentionally harnessed to control fluids and particles at the microscale. We first summarize the key materials and fabrication techniques for deformable devices. We then discuss how structural deformation can be exploited to enable various fluidic operations, as well as particle manipulation functions. Subsequently, we highlight representative applications that leverage device deformability in biomedicine and industry. Finally, we outline critical challenges and propose future research directions of the field for advanced manipulation.

## Graphical abstract

Deformable devices enable fluid and particle manipulation modes that are inaccessible to rigid platforms. This review examines soft materials and architectures that translate controlled deformation into microscale fluid operations such as pumping, valving, mixing, and particle handling. By establishing deformation–function relationships, this paper highlights emerging biomedical and industrial applications and prospects for adaptive, material‐enabled flexible systems.

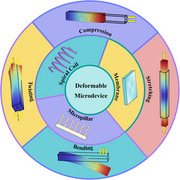

**Abbreviations**
Abbreviations: Full term
3D: three‐dimensional
APTES: (3‐aminopropyl) triethoxysilane
Au: gold
C–N: carbon–nitrogen
COC: copolymer
CTC: circulating tumor cell
CVD: chemical vapor deposition
DIW: direct ink writing
DLD: deterministic lateral displacement
*E. coli*
: *Escherichia coli*
ECTMS: 2‐(3,4‐epoxycyclohexyl) ethyl trimethoxysilane
EPV: elastomeric pinch valve
EpCAM: epithelial cell adhesion molecule
ESP: elastomeric suction pump
FDM: fused deposition modeling
CAD: Computer‐aided design
FN: fluid‐net
hMSC: human mesenchymal stem cell
LGEPM: liquid‐gating elastomeric porous membrane
LM: liquid metal
MPTMS: 3‐(mercaptopropyl) trimethoxysilane
MRE: magnetorheological elastomer
MR: magnetorheological
Ni: nickel
PDMS: polydimethylsiloxane
PI: polyimide
PLA: polylactic acid
PMMA: ploymethyl methacrylate
PPy: polypyrrole
PTFE: polytetrafluoroethylene
PVDF: polyvinylidene fluoride
Roll‐to‐roll: R2R
SLA: stereolithography
TiN: titanium nitride
TPE: thermoplastic elastomer
TPP: two‐photon polymerization
TPU: thermoplastic polyurethane
UV: ultraviolet

## 1 Introduction

Fluids and particles are both fundamental forms of matter. Fluids are substances that can flow and continuously deform under a shear stress such as liquids and gases, and particles are small, discrete solid or liquid bodies that may move or interact within a fluid medium such as cells and droplets [1, 2]. Fluid and particle manipulation are essential tasks in microfluidics, robotics, and biomedical engineering [3, 4, 5]. These tasks contribute to broad applications such as drug delivery [6], materials synthesis [7], and cell sorting [8]. Traditional microdevices are commonly fabricated by rigid materials (e.g., metal, glass, silicon, and hard polymer blocks) [9, 10], which lack mechanical flexibility to adapt to conformal contact and dynamic interaction with soft or irregular environments and the confined three‐dimensional (3D) spaces such as required by wearable and implantable devices [11, 12, 13]. Moreover, once a rigid device is fabricated, its geometries are fixed, resulting in limited tunability of fluid flow range and applicable target particle sizes [14, 15]. Iterative design, fabrication, and optimization are usually needed, significantly increasing the cost and time of research and development. Moreover, the rigid devices lack the flexibility to dynamically tune microchannel dimensions to control the kinematics and dynamics of fluids and suspended particles, resulting in limited functionality.

Deformable and flexible microdevices consist of soft materials and components, which can offer improved flexibility, functionality, and performance through device deformation [16]. According to the degree of deformability, these devices can be categorized as partially deformable, where only a portion of the device components can deform, and wholly deformable, where the whole device body can be deformed to achieve the function. Materials and fabrication methods are essential for the development of deformable devices. Various soft polymers such as silicon‐based polymers [17, 18], hydrogels [19], and thermoplastics [14] have been used for deformable devices. Meanwhile, fabrication techniques, including soft lithography [20, 21], additive manufacturing [22, 23], and hot embossing [24], as well as various bonding methods [25, 26, 27] can pattern high‐resolution small features on a layer and bond multiple layers together to construct deformable devices.

Controlling device deformation allows for on‐demand changes of device structures and dimensions, fluid behavior, and associated particle dynamics. Specifically, in fluid manipulation systems, the microchannels, membranes, or microstructures are deformed to regulate the duct opening and closing [34, 51, 52], flow speed [53, 54], and the spatial distribution of the fluids [35, 55]. In addition, shape‐morphing microchannels and microstructures can dynamically control hydrodynamic and mechanical forces acting on particles, enabling size‐tunable focusing [15, 39], sorting [40, 56], and controlled deformation of particles [42, 46]. The superior adaptability and versatile functionality of deformable devices have led to unique applications in biomedicine and industry. For instance, it has been successfully used in organ‐on‐a‐chip systems, where soft membranes and microstructures can transmit deformation to cells in vitro, thereby replicating mechanical stimuli of the physiological environment [42, 57]. In flexible sensors, the soft substrate can closely fit the human body shape to monitor the biophysical signal through sensing body motion and analyzing biofluids [49, 58, 59]. Moreover, the on‐demand changes of device structures through fluid‐solid interaction have been proven effective in soft robotics, where fluid‐driven compliant actuators are capable of bending, elongating, or twisting to perform gripping and crawling motions [50, 60]. Although there have been many reviews about flexible microfluidics [61], flexible electronics [62], and soft robotics [63], these reviews mostly focus on each specific area and lack a more unified overview. There is a lack of a unified framework in the current literature to link material and device deformability with operational mechanisms for fluid and particle manipulation at the small scale.

**FIGURE 1:**
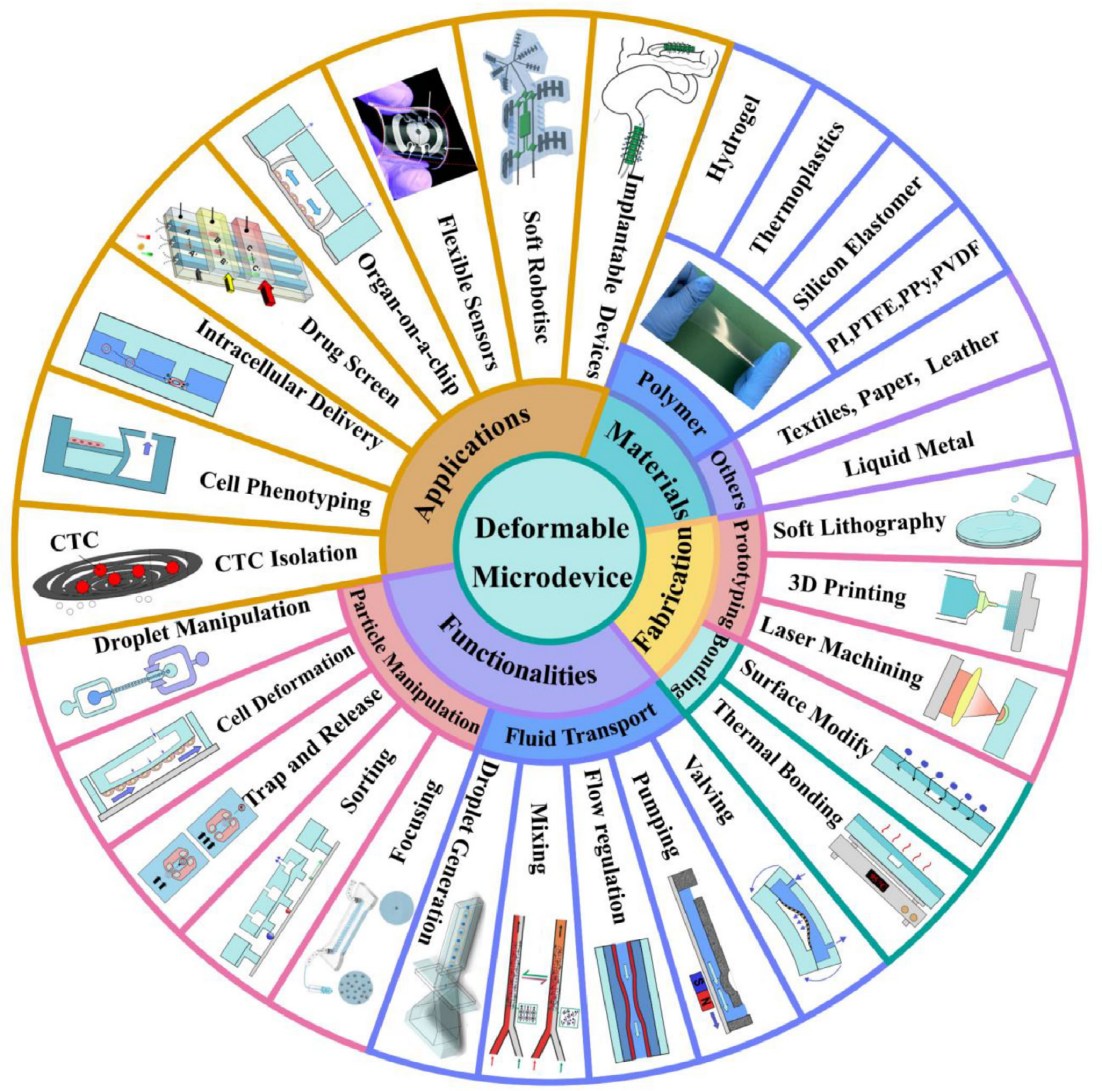
A schematic overview of engineering deformable devices, covering materials, fabrication methods, functionalities, and applications for fluid and particle manipulation. Polyimide (PI), polytetrafluoroethylene (PTFE) [28], polypyrrole (PPy) [29], polyvinylidene fluoride (PVDF), circulating tumor cell (CTC). Soft lithography. Reproduced with permission [16]. Copyright 2024, Royal Society of Chemistry. 3D printing. Reproduced with permission [30]. Copyright 2022, John Wiley and Sons. Laser machining. Reproduced with permission [31]. Copyright 2018, Elsevier. Surface modify. Reproduced with permission [32]. Copyright 2022, MDPI. Thermal bonding. Reproduced with permission [33]. Copyright 2023, MDPI. Valving. Reproduced with permission [34]. Copyright 2013, Royal society of chemistry. Pumping. Reproduced with permission [35]. Copyright 2017, John Wiley and Sons. Flow regulation. Reproduced with permission [36]. Copyright 2015, Royal Society of Chemistry. Mixing. Reproduced with permission [37]. Copyright 2015, Royal Society of Chemistry. Droplet generation. Reproduced with permission [38]. Copyright 2025, Elsevier. Focusing. Reproduced with permission [39]. Copyright 2024, AIP Publishing. Sorting. Reproduced with permission [40]. Copyright 2014, Elsevier. Trap and releasing. Reproduced with permission [41]. Copyright 2019, Elsevier. Cell deformation. Reproduced with permission [42]. Copyright 2023, Elsevier. Droplet manipulation. Reproduced with permission [43]. Copyright 2025, Springer Nature. CTC isolation. Reproduced with permission [44]. Copyright 2020, AIP Publishing. Cell phenotyping. Reproduced with permission [45]. Copyright 2019, Royal Society of Chemistry. Intracellular delivery. Reproduced with permission [46]. Copyright 2023, Royal Society of Chemistry. Drug screen. Reproduced with permission [47]. Copyright 2019, National Academy of Sciences. Organ‐on‐a‐chip. Reproduced with permission [48]. Copyright 2021, American Chemical Society. Flexible sensors. Reproduced with permission [49]. Copyright 2017, John Wiley and Sons. Soft robotics. Reproduced with permission [50]. Copyright 2020, John Wiley and Sons. Implantable devices. Reproduced with permission [13]. Copyright 2021, Springer Nature.

This review aims to provide a systematic study of how the device deformability can be intentionally used for fluid and particle manipulation, as shown in Figure 1. First, we summarize the materials and the fabrication techniques for the development of deformable microdevices. Next, we elaborate on how to take advantage of device deformability for several fundamental functions of fluid and particle manipulation. Subsequently, we summarize the successful applications through exploiting device deformability in biomedicine and industry, such as the isolation of circulating tumor cells, organ‐on‐a‐chip, and soft robotics, etc. Finally, we examine the key challenges and outline future perspectives for unlocking the full potential of deformable devices.

## 2 Materials for Deformable Devices

Deformable devices achieve their functionalities by tuning the geometry of the device body partially or entirely. Therefore, at least one component of the device should be made of flexible material. It is extremely critical to select suitable materials for the device [64]. This section focuses on the discussion of materials, primarily soft polymers, that enable micro components to deform. Table 1 summarizes the basic mechanical properties of these polymers.

**TABLE 1:** Properties of the main polymeric materials for deformable devices.

Materials	Type	Maximum strain	Young's modulus (MPa)	Advantages	Limitations	Refs.
PDMS	Silicone elastomer	100%–300%	1–3	High optical transparency, durability, biocompatibility, plasma bonding	Hydrophobic, limited strain, incompatibility with organic solvents	[67, 77, 157, 158, 159, 160, 161]
Ecoflex	Platinum‐catalyzed silicone	500%–900%	0.05–0.1	High flexibility, large elongation, biocompatibility	Inapplicable for plasma bonding, translucent, high viscosity	[68, 82, 87, 158]
Parylene C	Thermoplastic polymer	200%	2.758	Biocompatibility, chemical inertness, high transparency, excellent water and gas barrier properties	Inapplicable for plasma bonding, costly	[104, 162]
PMMA	Thermoplastic polymer	5%–20% (ultrathin)	2000–3200	Biocompatibility, high optical transparency, low reagents consumption	Low chemical resistance Inapplicable for plasma bonding,	[45, 46]
Flexdym	Thermoplastic elastomer	720%	1.18	Biocompatibility, high optical transparency, low sweat absorption and water vapor transmission rate	Difficult to achieve permanent covalent bonding	[14, 114, 115, 163]
Elastollan	Thermoplastic polyurethane	893%	2.4	High flexibility, good mechanical strength, chemical resistance, high optical transparency	Hygroscopic nature	[114, 158]
Polyimide	Thermosetting polymer	72%	3500	Biocompatibility, high thermal stability, good sealing properties, good mechanical strength	Opaque, Inapplicable for plasma bonding	[164]
Poly(N‐isopropylacrylamide) (PNIPAM)	Hydrogel	600%	0.035–0.105	Biocompatibility, structural tunability, tunable physical properties, high optical transparency	Low mechanical strength, dehydration in dry environments	[165, 166]

### 2.1 Polymeric Materials

#### 2.1.1 Polydimethylsiloxane (PDMS)

PDMS is an elastomeric polymer containing carbon and silicon, with the advantages of biocompatibility, mechanical robustness, corrosion resistance, easy fabrication, and excellent optical transparency [65, 66]. The cured PDMS elastomer (base‐to‐curing agent ratio of 10:1) has a Young's modulus of 1–3 MPa and a shear modulus of 0.4 to 0.9 MPa, making it promising for making flexible and deformable structures [67, 68]. The flexibility of PDMS can be tuned by the thickness [69] or the ratio between monomer and curing agent [68]. The ease of fabrication using replica molding or soft lithography makes PDMS the most popular material for deformable microdevices [20]. Besides molding, other fabrication methods for PDMS have been reported, including additive manufacturing [70], laser ablation and cutting [71], and spin coating [72], etc. PDMS exhibits prominent bonding performance and can be permanently bonded to itself or glass via plasma treatment [73]. To date, PDMS has been used as membranes [74], device bodies containing microchannel networks [75], and micropillars [76] to enable various device tasks.

However, PDMS also has some drawbacks. PDMS suffers from a hydrophobic nature due to its methyl (CH_3_) group [77]. This property hinders PDMS applications in cell culture and organ‐on‐a‐chip [78]. Oxygen plasma treatment [79] and surface coatings [80] can mitigate this drawback. Besides, PDMS is highly permeable and swellable in organic solvents such as toluene, chloroform, hexane, acetone, and ethanol, which could alter channel dimensions and device integrity [81]. Moreover, the limited strain range of PDMS restricts its application where a large elongation ratio is needed [14, 18, 82].

#### 2.1.2 Ecoflex and Dragon Skin Silicon Elastomer

Ecoflex [83] and Dragon Skin [84] are both platinum‐catalyzed liquid silicone rubbers made by Smooth‐On, primarily used for creating flexible, biocompatible molds and prosthetics [85]. These silicon elastomers are characterized by high flexibility, large elongation, and low Young's modulus (0.05–0.5 MPa) [86]. Their maximum strain can reach up to 1000%, making them popular in devices with large deformation and ultrastretchability [68]. Compared to Ecoflex, Dragon Skin exhibits a higher tensile strength and tear strength, making it suitable for components that require high mechanical performance [87]. In addition, most of Ecoflex and Dragon Skin are translucent [86], and only specific products such as Near Clear (e.g., Ecoflex 00–31 Near Clear and Ecoflex 00–45 Near Clear) offer better transparency, making them suitable for optical access inside the device [88].

Soft lithography [60], spin coating [89], and direct ink writing (DIW) [90] have been used to fabricate Ecoflex/Dragon Skin microstructures or membrane using their liquid‐state and room‐temperature curing properties. However, the presence of uncross‐linked silicone oil on these material surfaces hinders the effectiveness of plasma bonding [87]. Bonding strategies such as utilizing silicone adhesive (Sil‐Poxy) [91] or an uncured polymer solution as glue [50] can partially solve this issue, but these methods exhibit low bonding strength and are unsuitable for bonding elastomer slabs containing shallow microchannels and structures.

#### 2.1.3 PDMS–Ecoflex /Dragon Skin Hybrids

The hybrids of PDMS and Ecoflex or Dragon Skin have been developed to combine the excellent flexibility of Ecoflex/Dragon Skin and the bonding capability of PDMS. The PDMS(Sylgard‐184)‐Dragon Skin (Dragon Skin 0020) composite with a ratio of 1:3 exhibits a maximum strain of 230% and is capable of plasma bonding [92]. Another study shows that the plasma bonding threshold of PDMS (Sylgard‐184)‐Dragon Skin(Dragon Skin 10‐slow) hybrids is 1:7 [87]. The optical transparency of hybrids elastomer increases with the ratio of PDMS in the hybrids [93]. Similar to PDMS, Ecoflex, and Dragon Skin, the soft lithography and spin coating methods are applicable for the hybrid materials [94]. Overall, these properties enable the hybrid materials to fabricate devices with tunable mechanical strength and complex small structures that can withstand large elongations.

#### 2.1.4 Thermoplastics

Thermoplastic polymers soften when heated and harden when cooled, without undergoing any permanent chemical change [95]. Polymethyl methacrylate (PMMA) is a thermoplastic polymer known as acrylic or Perspex. PMMA is widely used in microfluidics due to its high transparency and biocompatibility [96]. Laser cutting [97], hot embossing [98], and micromilling [99] are the common fabrication methods for PMMA‐based devices. Thermal bonding [100], adhesive bonding [101], and surface modification bonding [102] are used to bond PMMA with itself or other materials. Due to its rigid characteristics, PMMA typically serves as the supporting layer for devices with no deformation. However, ultrathin (180–280 nm) PMMA film can have a maximum strain of ∼20% and can be employed as a deformable channel layer [103]. Parylene is another thermoplastic polymer with the advantages of biocompatibility, high transparency, flexibility, corrosion‐resistance, and excellent water and gas barrier [104]. Dry etching [105] and chemical vapor deposition (CVD) [106] are its common fabrication methods. The bonding strategies of Parylene are the same as those of PMMA [107]. Due to the high flexibility (200% maximum strain) and chemical inertness, Parylene has been employed as microchannels [108] and membranes [109] in deformable devices.

Thermoplastic elastomer (TPE) is a class of soft thermoplastic polymers that combines the elasticity of rubber with the processability of thermoplastics [110]. TPEs exhibit excellent flexibility at room temperature and can be easily reshaped at high temperatures [111]. Flexdym [112] is a commercial TPE with excellent optical transparency and flexibility (720% maximum elongation), low vapor transmission, and good biocompatibility [24]. Flexdym is an ideal material for a microfluidic channel due to its excellent sealing capability and low absorption of analytes [113]. Hot embossing is the common method to pattern microstructures and microchannels with Flexdym substrate [114]. Flexdym can form reversible spontaneous formation of bonds with diverse substrates (e.g., Flexdym, glass, PMMA) [24, 112]. The bonding strength can be enhanced by properly increasing the bonding temperature or performing surface modification such as plasma activation [115]. Thermoplastic polyurethane (TPU) is another relevant TPE. Elastollan is a commercial TPU brand from BASF that exhibits excellent flexibility (maximum strain of 893%) and chemical resistance [116]. Elastollan shows great potential for melt‐based fabrication processes, including 3D melt blowing [117] and fused deposition modeling (FDM) [118]. Elastollan has been used to fabricate membranes [119] and microstructures (e.g., scaffolds [120]).

#### 2.1.5 Hydrogels

Hydrogels consist of hydrophilic polymer networks that can store a large amount of water [121]. Hydrogel offers advantages such as biocompatibility, high deformability, and tunable physical properties [122]. The stretchability of hydrogel is excellent, and the reported maximum tensile strain can reach up to 2100% [123]. Additive manufacturing [124], soft lithography [125], and sacrificial template replication [126] are commonly used to fabricate microchannels and microstructures on hydrogels. To bond a hydrogel with other materials, an adhesive layer can be used [127], or an uncross‐linked hydrogel precursor can be applied between the materials to achieve twice‐cross‐linking [125]. However, pure hydrogel exhibits poor mechanical strength, and the microchannels made of hydrogels are prone to collapsing and deforming [128]. Hydrophilic polymer such as alginate can be added to the matrix to improve the mechanical strength of hydrogels [129]. Moreover, hydrogel faces shortcomings such as water absorption [130] and easy dehydration [131], limiting its extensive use.

Stimuli‐responsive hydrogels are a class of novel hydrogel materials [132] that undergo shape changes or sol–gel phase transitions when exposed to external stimuli such as temperature [19], pH [133], and solution concentration [134]. This unique property has been used to fabricate a shape‐tunable device for fluid [135] and particle manipulation [133]. In addition, the conductive hydrogel shows excellent electrical conductivity, which is promising for wearable electronics [136].

#### 2.1.6 Other Polymers

PI is another polymer extensively used in deformable engineering devices. PI offers advantages including great biocompatibility, excellent thermal stability (up to 400°C), and good mechanical strength [137]. Vapor deposition [138], laser micromachining [139], and etching [140] have been employed to fabricate PI membranes and microchannels. Chemical activation bonding and adhesive bonding are widely used to bond PI with itself or other materials [141]. Furthermore, various other polymers such as polytetrafluoroethylene [28], polypyrrole [29], and polyvinylidene fluoride [142] have been applied in deformable devices.

### 2.2 Nonpolymeric Materials

In applications where transmission of electrical signals is needed such as in flexible electronics, conductive materials are indispensable. Metals such as gold (Au) [143], nickel (Ni) [144], along with metal compounds titanium nitride (TiN) [145, 146] have been employed as thin films to accommodate deformation in devices. Liquid metal (LM) has both metallic and fluidic properties at room temperature [147], and is an emerging conductive material in flexible electronics. Gallium and its alloy (EGaIn, Gailinstan) are the most widely used LM, providing an excellent combination of conductivity and deformability [148]. LM can also be encapsulated within a soft microfluidic channel to serve as a sensor. The change in cross‐sectional area and the length of the channel can result in measurable variations in electrical resistance [49, 149].

Magnetic materials can be in the form of dispersed microparticles, such as carbonyl iron powder [35] and neodymium‐iron‐boron [150], which can be doped within flexible materials (e.g., PDMS) to make a magnetic actuated micropillar and membrane [35, 151]. Glass [152] and silicon [153] are rigid and have been used as the supporting layer for deformable devices such as micropumps and microvalves. Furthermore, other materials such as textiles [154], paper [155], and leather [156] also serve as available materials for deformable devices.

## 3 Fabrication Methods for Deformable Devices

### 3.1 Prototyping Techniques

Prototyping techniques refer to transferring the micropattern or microstructures onto the surface of soft polymers. Selecting suitable prototyping methods based on material type and specific microfeature is critical, as it directly influences the resolution, mechanical properties, and ultimately functionalities of devices [167]. In this section, we describe the common prototyping techniques with emphasis on their working principles, advantages, limitations, and applications.

#### 3.1.1 Photolithography

**FIGURE 2:**
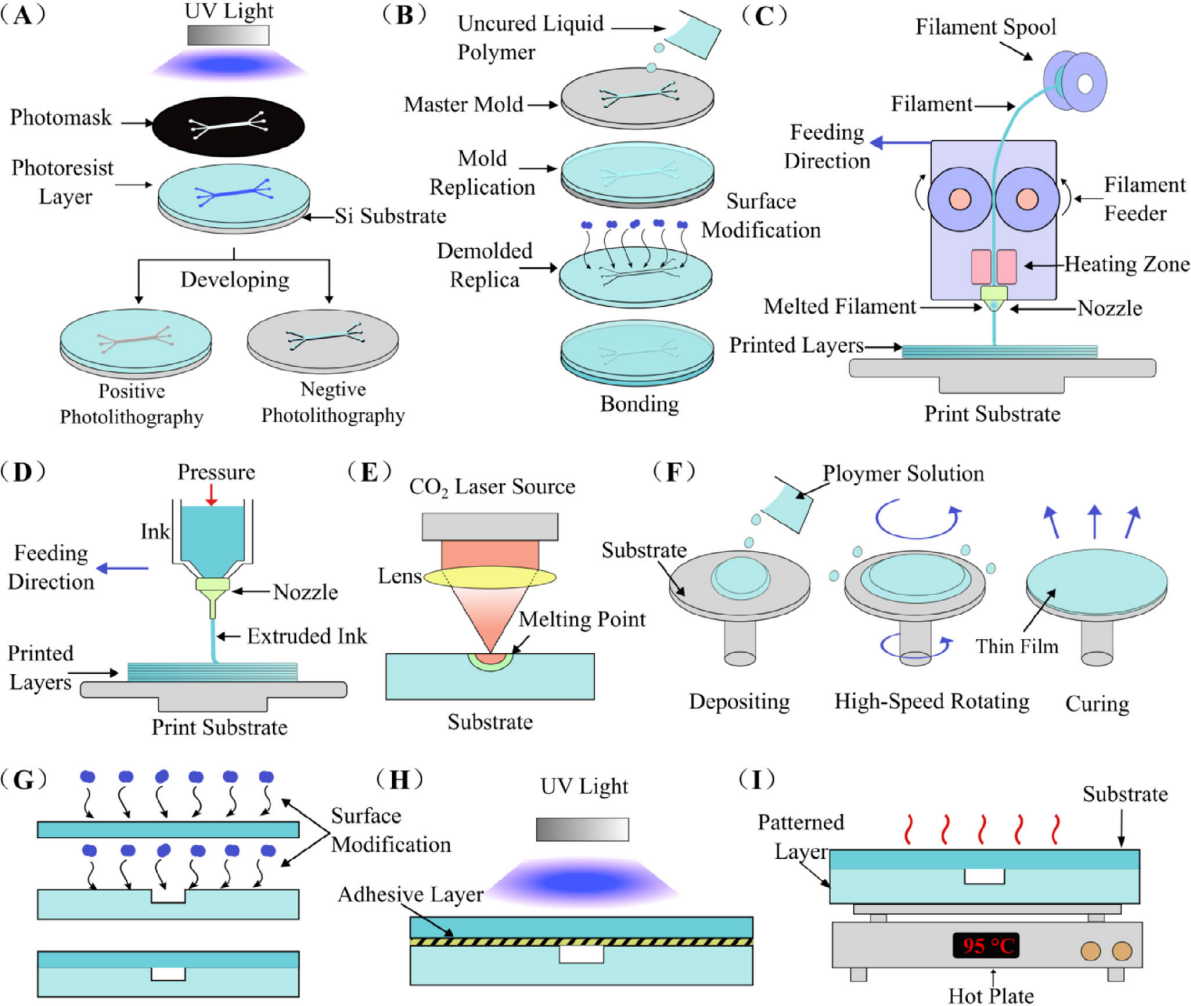
Deformable microdevice prototyping and bonding techniques. (A) Photolithography. Reproduced with permission [16]. Copyright 2024, Royal Society of Chemistry. (B) Soft lithography. Reproduced with permission [16]. Copyright 2024, Royal Society of Chemistry. (C) Fused deposition modelling. Reproduced with permission [201]. Copyright 2025, John Wiley and Sons. (D) direct‐ink‐writing. Reproduced with permission [30]. Copyright 2022, John Wiley and Sons. (E) Laser micromachining. Reproduced with permission [31]. Copyright 2018, Elsevier. (F) Spin coating. Reproduced with permission [202]. Copyright 2024, American Chemical Society. (G) Surface modification bonding. Reproduced with permission [32]. Copyright 2022, MDPI. (H) Adhesive bonding. Reproduced with permission [203]. Copyright 2008, Springer Nature. (I) Thermal boning. Reproduced with permission [33]. Copyright 2023, MDPI.

Photolithography and soft lithography are the most popular prototyping methods in microdevices due to their high resolution and compatibility with various materials [20, 21]. Photolithography leverages ultraviolet (UV) light exposure to transfer micropatterns from a photomask onto a photoresist coated on a substrate, Figure 2A [168]. After exposure, the part of the photoresist layer becomes dissolvable and can be removed by a developer solution [169]. Based on the type of photoresist used, photolithography is classified into positive and negative lithography [170]. The exposed region is removed in positive lithography. In contrast, the negative lithography dissolves the unexposed region [171]. Su‐8‐based negative lithography is widely used in the fabrication of microchannels and microstructures for microfluidics devices, or master molds for soft lithography [172]. Photolithography is compatible with many other processes, such as etching, sputtering, and lift‐off to fabricate a flexible microfilter [173] and electrode [174]. However, photolithography for the fabrication of microchannels and microstructures is generally an expensive and complex process [20].

#### 3.1.2 Soft Lithography

Soft lithography, replica molding, or simply molding provides an easier and more cost‐effective way by directly replicating geometries from a master mold or stamp, Figure 2B [175]. This process begins with the fabrication of a master mold, most commonly achieved by photolithography owing to its high precision [176]. Alternatively, methods such as 3D printing [177], laser micromachining [51], and casting [178] can also be employed for mold fabrication. Subsequently, uncured liquid materials (such as PDMS) are poured onto the patterned mold [179]. After complete curing and solidification of materials, negative microchannels and microstructures are formed by peeling off from the module [15] or dissolving the module [178]. Finally, a bonding process is required to encapsulate the microchannels and microstructures, enabling specific functionalities [179]. However, soft lithography includes several manual operations, such as manual pouring and bonding alignment, which decrease its precision and efficiency [180, 181].

#### 3.1.3 Additive Manufacturing

Additive manufacturing is also known as 3D printing. Unlike traditional methods that remove material from a substrate, additive manufacturing creates 3D objects by stacking materials layer by layer based on a computer‐aided design (CAD) model [182]. Additive manufacturing has the advantages of less material waste, automatic, and template‐free [183]. Sealed devices with complex geometries can be fabricated by 3D printing in a single step, without bonding processes [184]. However, compared to lithography, 3D printing suffers from low resolution and poor surface quality [185].

The commonly used 3D printing methods in deformable devices include FDM [23], DIW [22], and stereolithography (SLA) [186]. FDM heats a thermoplastic filament at the nozzle to a semi‐liquid state and deposits it onto the substrate or the previously printed layers [187], Figure 2C. The thermoelectricity of the polymer enables the filament to fuse together and form a structure during printing [188]. FDM is widely used in polymers such as thermoplastic TPU [118], polylactic acid (PLA) [189], and other TPEs [190]. However, the adjacent layers are poorly fused due to the rapid solidification of the extruded material, resulting in poor sealing performance of the device [191].

DIW constructs 3D geometries by extruding uncured ink, such as hydrogel [192] and PDMS [193] layer‐by‐layer through a dispenser nozzle, Figure 2D. Unlike the FDM, DIW is not temperature dependent; it leverages the rheological properties such as shear shinning behavior and viscosity to finish fabrication [194]. Therefore, the ink formulation and the optimization of printing parameters are critical for device fabrication using DIW [195]. SLA utilizes an energy source (light or electron beams) to initiate polymerization and solidify the liquid monomers [196]. Initially, this method was commonly used for resins and ceramics, but is now also used for hydrogels [135] and PDMS [197]. SLA provides high resolution at a relatively low cost [198], but it suffers from long processing time [196]. In addition to directly forming the structures of devices, 3D printing can also be used to make a sacrificial mold [199] or the master mold for replica molding [200].

#### 3.1.4 Laser Micromachining

Laser micromachining selectively removes, patterns, or modifies materials by concentrating energy at the focal point of the laser beam [204], Figure 2E. Laser micromachining is promising for the fabrication of a deformable device due to the advantages of noncontact, maskless, easy to operate, and high efficiency [205]. Commonly used laser sources include the CO_2_ laser [31], femtosecond laser [206], and UV laser [207]. This technique enables precise operations such as cutting [208], drilling [209], and welding [210]. Laser micromachining has been employed to fabricate a microfluidic channel by cutting through a thin polymer sheet, which is further enclosed by sandwiching between two supporting layers [211]. In addition, microfilters [209], fluidic chambers [51], and other microstructures [212] can also be formed through laser micromachining. However, the laser‐machined surfaces are prone to defects such as burrs or microcracks due to the high thermal load [213].

#### 3.1.5 Spin Coating

Spin coating is a widely used technique for producing thin uniform films from homogeneous polymer solutions [214]. Spin coating leverages centrifugal force and the surface tension of solutions generated by high‐speed rotation to deposit a uniform polymer film onto the substrate surface [202], Figure 2F. Films with specific thickness (ranging from a few nanometers to several micrometers) can be obtained by adjusting the rotation speed and duration [215]. To pattern microfeatures on the thin films, spin coating has to be integrated with other prototyping techniques, such as soft lithography [216] and laser direct writing [217] to selectively remove or modify the surface of the film. However, the material waste rate of spin coating is generally high (over 90%), indicating a high fabrication cost [218].

#### 3.1.6 Hot Embossing

Hot embossing is a common technique for patterning thermoplastic materials [24]. In hot embossing, thermoplastics are heated above their glass transition temperature (*T*_g_), where thermoplastic materials become softened and rubbery [219]. Meanwhile, thermoplastics are pressed against a master mold with positive microstructures and features, and the softened polymer flows and conforms to the mold's small features [220]. After cooling the polymer below *T*_g_, the patterned negative microfeatures remain on the polymer surface, following the release from the mold. Hot embossing has the advantages of high throughput and good surface quality [221, 222].

#### 3.1.7 Other Methods

Micromilling is a subtractive manufacturing technique that uses miniaturized rotating tools to remove material from a workpiece [223]. Micromilling is generally used to fabricate channels, holes, cavities, or molds for microstructure replication [224]. This method offers advantages such as automation, high resolution, and high efficiency. However, due to the low stiffness of soft polymers at room temperature, they need to be cooled to near their *T*_g_ (e.g., −123°C for PDMS) to enable precise micromilling [225, 226].

Sputtering utilizes energetic ions that bombard the conductive target material and deposit on the substrate [227]. Sputtering is commonly used to fabricate thin film conductive electrodes on a polymer substrate for flexible electronics [143]. Moreover, other prototyping methods such as chemical vapor deposition [228] and wet etching [229] have been used to fabricate deformable devices.

### 3.2 Bonding Strategies

In the above sections, we discussed the prototyping techniques to form micro/nanofeatures on the surface of a soft material. However, to achieve the functionality of devices and to protect fluidic samples, microstructures need to be enclosed within a membrane or a substrate. Therefore, robust and reliable bonding techniques are essential for the fabrication of deformable devices. An excellent bonding technique will prevent layer delamination and fluid leakage, provide structural stability and mechanical strength, enable 3D architectures, and integrate materials with different functions [230]. This section summarizes three main bonding methods in silicon‐based materials and thermoplastics.

#### 3.2.1 Surface Modification Bonding

Surface modification bonding leverages physical or chemical processes to remove surface contaminants and generate reactive chemical groups for covalent bonding, resulting in superior bonding strength [27], Figure 2G. Physical treatment employs UV/ozone [231] or plasma [232] to render material surface properties. Plasma treatment is the most commonly used bonding method for silicon‐based materials such as PDMS and glass [233]. During treatment, the terminal methyl groups (─CH_3_) on the PDMS surface are replaced with the silanol groups (─Si─OH), which enable covalent siloxane bonds (Si─O─Si) with another treated surface for permanent bonding [234, 235]. UV/ozone treatment offers the same bonding effects as plasma treatment, but with a slower rate [236]. In addition, physical surface treatment such as plasma treatment can also enhance the surface quality and the hydrophilicity of materials [231]. However, the surface energy provided by physical methods is insufficient to achieve permanent bonding for thermoplastic materials [237] and elastomers such as Ecoflex/Dragon Skin [87].

Chemical surface modification bonding utilizes chemical reagents to introduce functional groups onto material surfaces, enabling covalent reactions between these groups when these surfaces contact [238]. Commonly used silane coupling agents include (3‐aminopropyl) triethoxysilane (APTES) [239], 2‐(3,4‐epoxycyclohexyl) ethyl trimethoxysilane (ECTMS) [240], and 3‐(mercaptopropyl) trimethoxysilane (MPTMS) [241]. ECTMS‐treated PDMS can be permanently bonded to APTES‐treated thermoplastics through the formation of strong covalent carbon–nitrogen (C─N) bonds between the two surfaces [240].

#### 3.2.2 Adhesive Bonding

Adhesive bonding applies liquid glue or a dry adhesive layer between two bonding substrates to form a bond and is considered one of the simplest bonding methods [242], Figure 2H. Adhesive bonding works through a mix of mechanical interlocking, secondary interactions (van der Waals, H‐bonds), and sometimes covalent bonding [243]. The adhesive contains UV [244] or pressure‐sensitive [245] initiators; when exposed to UV light or pressure applied, the material forms adhesion between the layers [246]. This method has been widely used for bonding PDMS to silicon‐based material [247], PDMS to thermoplastics and other materials [246, 248], as well as thermoplastics to thermoplastics [101]. However, the bonding strength achieved by this method is generally lower than that of surface modification bonding [230].

Specialized solvents can dissolve the surface of the thermoplastics, forming a solvated layer that enables polymer chains to entangle upon contact [249]. For instance, acetic acid‐treated PMMA substrates can be bonded to each other by low‐pressure clamping at room temperature in only 30 s [250]. In addition, an uncured polymer solution [50] and hydrogel [125] can serve as glue to achieve strong bonding through the twice‐cross‐linking occurred during the glue curing process [251]. However, in adhesive bonding, the excessive liquid adhesive glue is prone to filling and clogging the microchannel and its structures [252].

#### 3.2.3 Thermal Bonding

Thermal bonding, also known as thermal fusion bonding, is commonly used in thermoplastic materials such as TPE and PMMA [25, 26]. In thermal bonding, polymer layers are heated above their *T*_g_ and pressed together to form a strong and permanent bond [253], Figure 2I. However, the rapid decrease in elastic modulus above *T*_g_ and the application of excessive pressure during the heating process can lead to deformation of the microchannel and microstructure [203]. Surface treatment such as plasma and UV modification has been employed to decrease *T*_g_ for thermal bonding, so that thermoplastic layers can be effectively bonded at temperatures lower than the *T*_g_ of the bulk polymer [254]. The *T*_g_ of copolymer (COC, *T*_g_ = 108°C) is reduced to 75°C after O_2_ plasma treatment. Moreover, the higher surface energy after treatment allows it to be bonded with PMMA (*T*_g_ = 105°C) at temperatures below 75°C with high bonding strength [255].

## 4 Functionality of Deformable Devices

### 4.1 Fluid Manipulation

Fluid manipulation controls the opening and closing of the fluid duct, the flow direction and speed, the spatial distribution of fluids, and pinching‐off of continuous streams into discrete liquid segments, which corresponds to core functions of valving [34, 51, 209, 257], pumping [54, 258, 259, 260, 261], flow regulation [53, 258, 261], mixing [35, 37, 55], and multiphase droplet generation [17, 38, 262, 263]. Soft materials such as PDMS, thermoplastics, and hydrogel have been widely employed in microdevices, which enable and improve fluid manipulation functionalities by deforming either specific components and regions [35, 37, 51, 257, 259] or the entire device body [34, 38, 55, 209, 261]. This section elaborates on the recent advances in using device flexibility to facilitate fluid manipulation in these functionalities.

#### 4.1.1 Valving

Valving is one of the most critical and basic functionalities in fluidic microdevices. Microvalve precisely controls the opening and closing of the microchannel and regulates both the flow direction and resistance [264]. Deformable microvalves function by morphing the membrane, channel, or the entire device body. Compared to traditional rigid microvalves, deformable valves take advantage of compactness, less leakage, and ease of actuation [11, 51].

**FIGURE 3:**
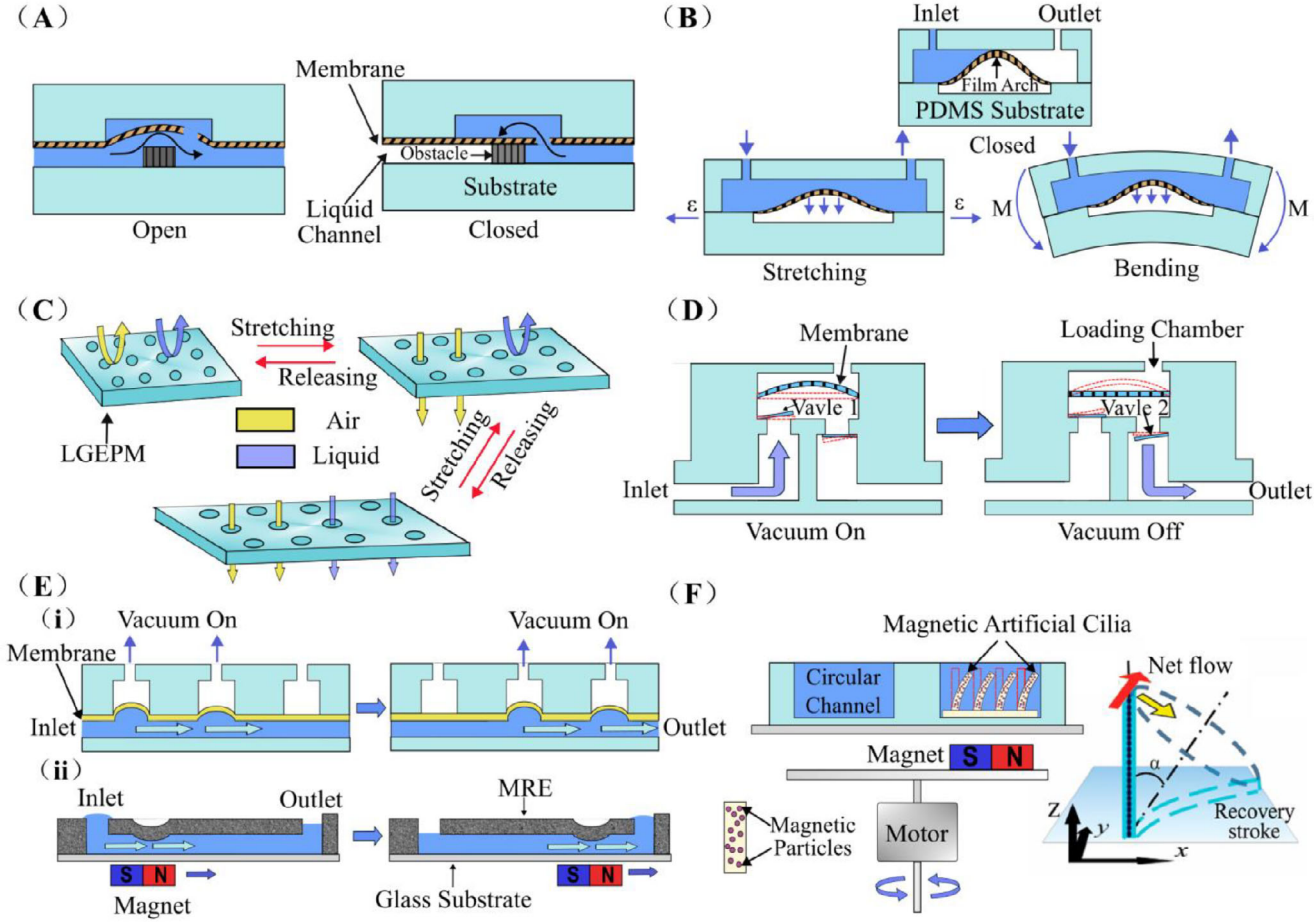
Enabling fluid valving and pumping using device deformability. (A) Partially deformed check valve. Reproduced with permission [51]. Copyright 2018, Elsevier. (B) Entirely deformed microvalve. Reproduced with permission [34]. Copyright 2013, Royal society of chemistry. (C) Liquid‐gating elastomeric porous membrane (LGEPM). Reproduced with permission [209]. Copyright 2018, The American Association for the Advancement of Science. (D) Pneumatic micropump. Reproduced with permission [54]. Copyright 2024, Royal society of chemistry. (E) Peristaltic micropump actuated by (i) pneumatic pressure and vacuum. Reproduced with permission [256]. Copyright 2000, The American Association for the Advancement of Science. (ii) Magnetic force on magnetorheological elastomer (MRE). Reproduced with permission [35]. Copyright 2017, John Wiley and Sons. (F) Magnetic artificial cilia micropump. Reproduced with permission [151]. Copyright 2018, Elsevier.

Based on the energy source used, the microvalve can be divided into passive and active valves [264]. The passive microvalves rely solely on the internal energy of the system (e.g., fluid flow or pressure), making it compact and easy to integrate with other subsystems [265]. A check valve (one‐way valve) is a common passive valve that allows fluid to flow in only one direction while preventing backflow [266]. For example, Figure 3A shows a thermoplastic check valve made by stacking TPU film and PMMA substrates [51]. Under forward flow, the membrane deforms once the liquid pressure exceeds a certain threshold, allowing the liquid to bypass the obstacle and flow through channels. While in reverse flow, the fluid enters the upper chamber through the hole, closing the valve and preventing the backflow. This check valve can withstand a maximum pressure of 30 psi for 24 h without any leakage.

Active microvalves take advantage of external energy to realize their valving functions [267]. Active microvalves can be actuated by pneumatic [257], mechanical [34], magnetic [268], and acoustic [269], and other external energy sources. Active microvalves include normally open or normally closed types based on their default state [52]. Pourmand et al. [51] developed a normally closed pneumatic microvalve that allows bidirectional fluid flow. The valve closes when a certain positive or atmospheric pressure is applied, and opens the flow under a negative pressure where the membrane deflects into the vacuum cavity. The experimental results show that the valve was leakage‐free when the positive pressure exceeded 5 psi and fully opened under a negative pressure of −3.5 psi. In these valves, only the membrane in the device deforms and rigid substrates are needed, which could hinder their integration with other deformable devices [270].

Meanwhile, the microvalves actuated through the deformation of the whole device body have also been reported. Figure 3B illustrates a normally closed, manually operated, entirely deformable microvalve based on PDMS [34]. To fabricate this device, a thin PDMS film is first bonded to a prestretched substrate. Releasing the substrate induces a buckled structure that serves as the internal valve. Upon manual stretching or bending, the valve opens as the arch's curvature decreases, allowing fluid to flow through. The valve returns to its original state once the external force is removed. In addition, Sheng et al. [209] proposed a dynamical gas–liquid transport microvalve, Figure 3C. The valving function is achieved by stretching an LGEPM. The LGEPM maintains a constant set point pressure *P*, which is the minimum pressure required to open the liquid‐gate. However, the critical pressure (*P*_c_) of gas and liquid, defined as the minimum pressure to push fluid through the membrane pores, decreases as stretching the membrane to enlarge the pore size of the LGEPM. If *P* exceeds the *P*_c_, the fluid can pass through the membrane. Stretching the membrane to adjust *P*_c_ so that *P*_c(gas)_ just falls below the set point pressure (i.e., *P*_c(gas)_ < *P* < *P*_c(liquid)_). At this point, only gas can pass through the membrane, while liquid cannot. In this way, gas and liquid can be separated.

#### 4.1.2 Pumping

Micropumps transport fluids in one direction and precisely adjust their flow rate and volume [271, 272]. Micropumps drive fluid flow by passive capillary force [273], osmotic pressure [274], gravity [275], or surface tension [276], or active controllable pneumatic [277], magnetic [35], and acoustic actuation [278] forces etc. Deforming a soft liquid chamber or channel can generate a pressure gradient to drive liquid movement. Generally, the combination with valves can ensure unidirectional flow [279]. Figure 3D depicts an active pneumatic valved micropump [54]. This micropump includes two soft one‐way valves, a pump membrane, and a pressure loading chamber. When a negative pressure is applied to the loading chamber, the membrane deflects, opening valve 1 while keeping valve 2 closed, allowing liquid to be drawn into the cavity. Under positive pressure, the membrane resets and the fluid is expelled through valve 2. Repeating these processes can pump fluids along a single direction. Moreover, manual‐powered [216], solenoid [280], and piezoelectric [281] actuators have been used in this kind of micropumps.

Peristaltic micropump is another common type of active pump, which generates net flow by sequentially deforming and releasing the microchannel [282]. They drive the fluid with a traveling wave without requiring rectification components such as valves, thus offering advantages such as simplicity and miniaturization [283]. A peristaltic micropump can be further categorized into continuous and discrete schemes [259]. The discrete scheme employs multiple actuators to deform the channel at multiple discrete locations perpendicular to the flow direction [256], Figure 3E(i). Three pneumatic control channels are positioned above the soft fluid channel, and pumping is achieved by opening these pneumatic channels in sequence. Besides, the camshaft [284], piezoelectric [285], and electrostatic [286] actuators have also been used in discrete peristaltic pumps. In contrast, in the continuous scheme, the actuator deforms microchannels continuously to drive the fluid along the channel direction. Figure 3E(ii) depicts a continuous peristaltic micropump utilizing an MRE microchannel [35]. A travelling magnet deforms the MRE and compresses the microchannel continuously along the flow direction, thus generating continuous pumping. However, the pumping efficiency of the peristaltic micropump is low due to the backflow without check valves [259].

In addition to the deformation of channels or chambers to pump the fluid flow, the periodic movement of microstructures inside the channel can also induce net fluid pumping. For example, a magnetic artificial cilia pump is composed of an array of magnetic elastomer pillars embedded within an open microchannel. The pillars are actuated by a rotating magnet below the device [151], left of Figure 3F. Pumping is achieved by the net flow induced through the asymmetric beating of each pillar during the magnetic rotation cycle, right of Figure 3F [287]. Magnetic artificial cilia pumps have the advantages of low “dead volume” and excellent controllability [260].

#### 4.1.3 Flow Regulation

In addition to the above valving and pumping, precise control of flow properties, such as flow velocity change with time at a given location or flow response to pressure disturbances or fluctuations, is of great importance for microfluidic applications [288]. Conventional solution demands complex external equipment and control strategies [289]. In contrast, the flexibility of the membranes and softness of channels can be tailored for these functions in a more compact, simple, and cost‐effective manner [53, 261]. This section will mainly discuss three flow controlling and regulating devices: flow regulator, flow stabilizer, and flow oscillator.

**FIGURE 4:**
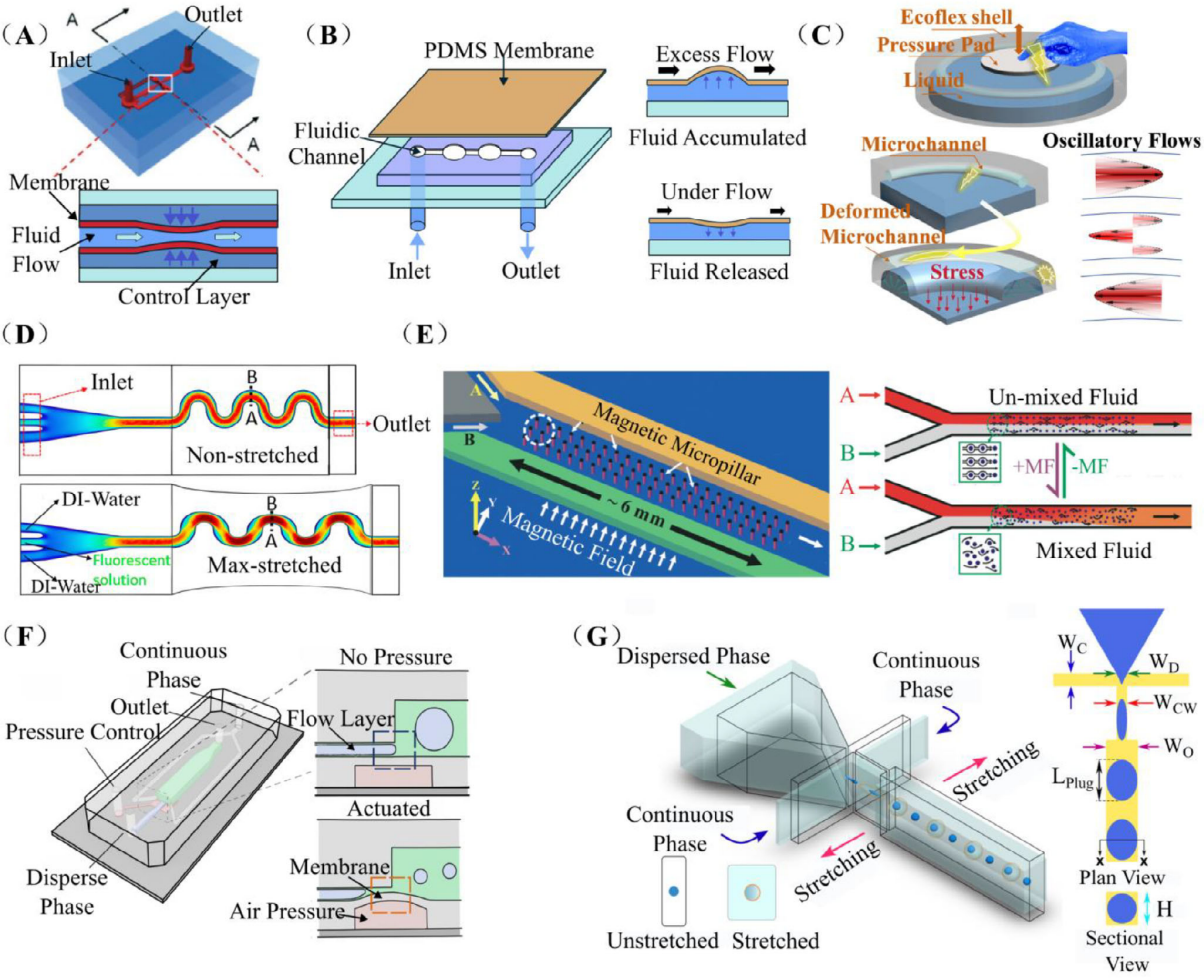
Facilitating fluid flow regulation, mixing, and droplet generation using device deformability. (A) Deformable flow regulator. Reproduced with permission [36]. Copyright 2015, Royal Society of Chemistry. (B) Compliant flow stabilizer. Reproduced with permission [258]. Copyright 2015, IOP Publishing Limited. (C) Entirely deformable flow oscillator. Reproduced with permission [261]. Copyright 2025, John Wiley and Sons. (D) Stretchable serpentine micromixer. Reproduced with permission [55]. Copyright 2021, Research Square. (E) Magnetic micropillar array micromixer. Reproduced with permission [37]. Copyright 2015, Royal Society of Chemistry. (F) Pneumatic actuated tunable step emulsification for droplet generation. Reproduced with permission [263]. Copyright 2024, Royal Society of Chemistry. (G) Stretchable flow‐focusing droplet tunable generator. Reproduced with permission [38]. Copyright 2025, Elsevier.

A flow regulator is a device that aims to precisely control and maintain a desired flow rate or pressure despite fluctuations [290]. Active flow regulators provide high‐throughput and broad flow rate adjustment, but complex design and bulky equipment are required [36]. Passive devices adjust the flow rate by changing the dimensions of the fluidic channels without external energy. Figure 4A shows a soft membrane‐based flow regulator [53]. This device consists of a main channel layer, two control channel layers, and two soft membranes. The main channel holds a contraction area to define the main flow resistance of the regulator. Once the pressure exceeds the threshold, the fluid is directed into the control channel and deflects the membrane to compress the main channel, increasing its resistance. The increased resistance compensates for the pressure increment, making the flow rate relatively constant.

Flow stabilizers are commonly used to reduce the fluctuations and smooth the flow, ensuring a steady, uniform output [291]. In practice, the pumping systems are never perfect and often experience disturbance due to equipment vibration, tubing compliance, or pressure fluctuations [75]. Flow stabilizers play an important role in applications where a stable flow is needed, such as chemical synthesis [292] and sample injection [293]. The flexibility and compliance of the membrane have been used to achieve flow stabilization. Figure 4B illustrates a passive compliant flow stabilizer [258], consisting of multiple compliant PDMS chambers above the fluid channel that function as fluidic capacitors. During the positive flow (overflow) cycle, the chambers expand to accumulate the excess fluid, whereas during the negative flow (underflow cycle), the membranes constrict to release the flow, thereby reducing the fluctuations and stabilizing the flow.

In contrast to a flow stabilizer, a flow oscillator aims to induce strong periodic variations in the fluid and convert the constant input into pulsatile output [294]. Conventional oscillatory flow generating systems are complicated [295]. Recent development has employed the flexibility of fluidic chambers and channels, Figure 4C [261]. The device consists of a central sealed liquid chamber enclosed in an Ecoflex shell and surrounded by a hollow microchannel. Periodic squeezing and releasing of the sealed chamber induce reciprocating fluid motion and oscillatory flow. In addition, magnetic artificial cilia array have also been used to create oscillatory flow [151]. Periodically reversing the rotation direction of the actuating magnet drives cilia to generate net flow in alternating directions, thus forming an oscillatory flow.

#### 4.1.4 Mixing

Fluid mixing refers to the process of homogenizing two or more miscible fluids, widely used in chemical and biomedical applications [296, 297]. Due to the small scale of the channel in microfluidics, the flow is normally within the laminar flow region. In rigid microchannels, the laminar nature of fluid flow leads to slow cross‐stream diffusion, which makes efficient fluid mixing challenging [298]. In contrast, the interactions between the soft channel walls and fluids in deformable microchannels can induce flow instability and chaotic advection, facilitating rapid mixing [299, 300]. In addition, utilizing the deformability of the whole device or partial components can enhance cross‐stream flow advection and instability. Figure 4D illustrates a stretchable micromixer consisting of serpentine channels [55]. The device is fabricated by stacking thin PDMS films, so that the whole device can be elongated. Periodically stretching the whole device can rapidly alter the channel length, cross‐section, and curvature. These changes lead to the transformation of Dean vortices and affect the mixing efficiency.

In contrast to the deformation of the whole device, fluid mixing can also be induced by partially deforming the device. A micromixer is comprised by a MRE cover and a PDMS circular chamber [35]. Periodical presence and absence of magnetic fields deflect the MRE cover repetitively, which induces the chaotic advections within the chamber and enhances the fluid mixing. Besides, a magnetic artificial cilia array inside a microchannel can also induce fluid mixing [37], Figure 4E. The micropillars remain upright and have limited impact on fluid mixing without the external magnetic field. Under a magnetic field, the pillars deflect and contact adjacent pillars randomly due to their small spacing. This disorganized configuration distorts fluid flow within the channel, thus improving the mixing effects. Reducing the gap between the pillars could further enhance the mixing performance.

#### 4.1.5 Droplet Generation

Droplets can be formed when two or more immiscible fluids (e.g., oil and water phases) are mixed [301]. In droplet microfluidics, an immiscible fluid stream could be pinched off by another due to the interplay of interfacial tension, viscous shear, and pressure forces, breaking into discrete droplets [302]. This technique has been widely used in single‐cell analysis [303], food industry [304], and drug delivery [6]. Droplet generation is significantly affected by the fluid properties (viscosity, interfacial tension), flow conditions (flow rate ratios, pressure), channel geometry and dimensions, etc. [38]. Therefore, modifying the channel geometry and dimensions can adjust the characteristics of the droplet generation process.

Dynamically deforming parts of a channel is a common strategy for controlled droplet generation. Figure 4F illustrates a tunable step emulsification generator based on pneumatic actuation [263]. Step emulsification drives a dispersed‐phase liquid from a narrow constriction to a wider chamber filled with continuous‐phase liquid, thus generating droplets due to the capillary pressure between the nozzle and the chamber. The size of droplets is mainly determined by the geometry of the nozzle, but the droplet size range is limited by adjusting the flow rates only. Pneumatically actuating a thin PDMS membrane beneath the emulsifying nozzle can reduce the height of the nozzle, decreasing the droplet size. Meanwhile, a similar approach has been used for a flow‐focusing configuration, where the continuous phase converges and breaks the dispersed phase into droplets at a narrow orifice [17]. A pneumatic balloon actuator at the orifice applies air pressure to narrow the orifice cross‐sectional area and increase shear, enabling smaller droplet formation without altering flow rates.

More recently, Roshan et al. introduced a fully stretchable droplet‐based microfluidic device [38], Figure 4G. The whole device is made of thin PDMS films. Lateral stretching enlarges the width of the dispersed phase inlet (*W*_D_), constriction (*W*_CW_), and droplet outlet channel (*W*_O_), while reducing the channel height (*H*) and the continuous phase channel width (*W*_C_). These geometric changes prolong the droplet detachment time and reduce the shear force, increasing droplet diameter and spacing and decreasing droplet generation frequency. Furthermore, the same strategy has been expanded to double emulsion generation, where multiple flow‐focusing junctions are connected in series [262]. This device consists of a two‐step flow‐focusing junction configuration. Precise control of the core size and shell thickness of double‐layered droplets becomes possible by selectively stretching the single junction unit or the whole device.

### 4.2 Particle Manipulation

Particle manipulation controls the position, motion, dynamics, and morphological properties of synthetic and natural particles [3]. Particle manipulation provides opportunities for both fundamental research and applications, such as materials synthesis [7], single‐cell analysis [41], and environmental monitoring [18]. Common particle manipulation methods include focusing [14, 15, 39], separation [18, 19, 40, 56, 133], trapping and release [41, 305, 306], mechanical deformation [42, 46, 307], and droplet merging and splitting [43, 308, 309]. With the integration of soft materials, shape‐morphing microchannels and size‐tunable microstructures can be fabricated to control the flow behavior, hydrodynamic force on particles, and particle‐wall interactions, thereby regulating the movement, trajectories, and deformation of particles. In this section, we will review the precise manipulation of particles through deformable structures.

#### 4.2.1 Particle Focusing

**FIGURE 5:**
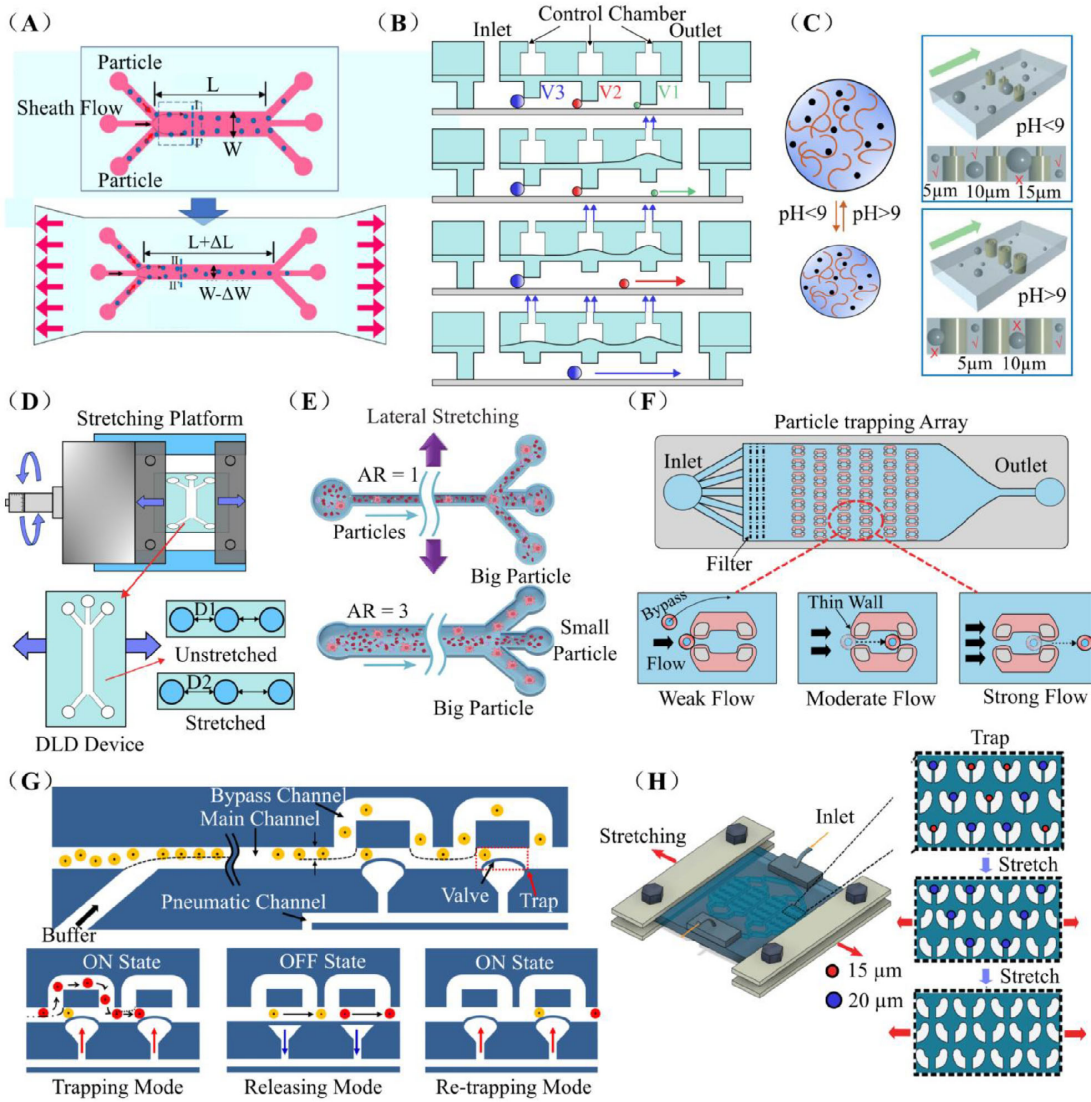
Exploiting device deformability for particle focusing, sorting, and trapping and release. (A) Stretchable inertial focusing device. Reproduced with permission [15]. Copyright 2020, American Chemical Society. (B) Multilevel pneumatic particle filter. Reproduced with permission [40]. Copyright 2014, Elsevier. (C) PH‐sensitive hydrogel particle filter. Reproduced with permission [133]. Copyright 2019, Royal Society of Chemistry. (D) Stretchable deterministic lateral displacement (DLD) device. Reproduced with permission [56]. Copyright 2008, Royal Society of Chemistry. (E) Stretchable viscoelastic particle sorting device. Reproduced with permission [14]. Copyright 2024, American Chemical Society. (F) Hydrodynamic‐driven deformable trapper array. Reproduced with permission [41]. Copyright 2019, Elsevier. (G) Bypass‐assisted pneumatic trapping device. Reproduced with permission [305]. Copyright 2013, Springer Nature. (H) Stretchable device for particle trapping and release. Reproduced with permission [306]. Copyright 2022, Royal Society of Chemistry.

Particle focusing refers to aligning particles into narrow streams or stable positions. This approach generally serves as a prior step to particle sorting, counting, and analyzing [310]. Fallahi et al. reported a stretchable inertial microfluidic device comprised of a straight rectangular channel and fabricated by thin PDMS films [15], Figure 5A. Stretching the straight channel in length can prolong particle migration time while reducing the lateral displacement required, thus promoting more effective migration and focusing at the channel center. The focusing efficiency (defined as the ratio of particle number in the equilibrium position to the total number) of 15 µm particles is 28.9% without stretching and increases to 100% at 6 mm stretching. Similarly, Liu et al. [39] developed an ultrastretchable viscoelastic particle focusing device made of Ecoflex. This device includes a circular channel and can be elongated up to 900% without failure. Elongation of the circular straight channel increases its length and reduces its radius, thus enabling the smaller particles and cells to focus on the channel centerline.

#### 4.2.2 Particle Sorting

Particle sorting aims to separate specific particles from mixtures [311]. Directly deforming the channel structure to create an adjustable filter gap is the most straightforward strategy for particle sorting. Figure 5B illustrates a deformable multilevel microchannels (MLMs) device for controlled particle sorting [40]. The device comprises three stepped filter gaps with decreasing gaps. The size of the gaps can be individually controlled by the pneumatic pressure in the cavity. Particles trapped at three filter gaps can be released sequentially by applying a vacuum to each pneumatic channel in order, thus realizing size‐based separation. In addition, stimuli‐response hydrogel pillars are promising for tunable particle filtration because their shape can reversibly change during phase transition [312]. Figure 5C shows a tunable particle filter consisting of a pH‐sensitive hydrogel micropillar array [133]. The micropillars swell when pH value is below 9, and vice versa, tuning the gap from 7.2 to 11.4 µm. Therefore, effective sorting of particles with different sizes can be achieved by dynamically adjusting the solution pH.

Deterministic lateral displacement is a passive sorting technique that directs particles to different streamlines in a micropillar array based on their size [313]. The trajectory of the particles is determined by the particle size and the critical diameter (*D*_c_) of the DLD device [314]. Particles smaller than *D*_c_ follow the flow direction, while particles larger than *D*_c_ move at an angle relative to the flow direction. Beech and Tegenfeldt [56] reported a stretchable DLD device for tunable separation of particles, Figure 5D. Laterally stretching the device increases *D*_C_ from 14.6 to over 16 µm, expanding its separation scope. Furthermore, PNIPAM hydrogel has been employed as a temperature‐controlled pillar geometry in a DLD device [19]. As the temperature rises from 20°C to 30°C, *D*_c_ increases from 1 to 10 µm, enabling separation across a wider range of particle sizes.

Meanwhile, stretchable inertial and viscoelastic microfluidic sorting devices have also been reported [14, 15, 18, 82]. In these devices, particles are first confined near the wall using a sheath flow, and particles migrate toward the channel center due to the inertial and or viscoelastic lift forces. Larger particles migrate faster than the small ones, therefore enabling size‐based separation [315]. The particle separation cut‐off size is highly dependent on channel length. Therefore, turning the channel length in real‐time can dynamically adjust the particle separation performance. For example, Yan et al. reported a sheath‐flow‐assisted ultrastretchable viscoelastic device for particle separation [18]. As the elongation increases from 0% to 300%, 20, 15, and 10 µm particles are separated and collected in the central outlet in sequence. In addition, varying the channel aspect ratio (defined as the ratio of channel width to height) through lateral stretching on devices can also modify particle sorting [14], Figure 5E. When the channel aspect ratio changes from 1 to 3, 15 µm particles migrate toward the side due to their high blockage ratio, whereas 4.8 µm particles still focus on the centerline, resulting in particle separation by differential equilibrium positions.

#### 4.2.3 Particle Trapping and Release

Particle trappers capture and immobilize individual particles at a predesigned location, followed by their controlled release after subsequent operations [316]. Particle trapping and release play a crucial role in downstream applications such as single‐cell analysis [317] and drug screening [41, 200]. Real‐time deformable trapping microstructures can enhance the trapping and releasing performance. Lee et al. [41] developed a device with a hydrodynamic‐driven deformable trapper array, Figure 5F. The front and rear gap of each trapper are thin and deformable by hydrodynamic force, with the rear gap narrower than the front gap. Under a moderate flow, particles are captured by the front gap. As the flow rate increases, the enhanced hydrodynamic force expands the front gap to release the particles, allowing them to enter the cavity and be retained at the rear gap. Finally, an even stronger stream deforms the rear trap sufficiently to release the particles completely from the trapper. In addition, Figure 5G illustrates a bypass channel‐assisted trapping device [305]. This device integrates multiple pneumatic membrane trappers and bypass channels to immobilize and release particles on demand. Pneumatic pressure controls the degree of membrane deformation, thus tuning the trapping size. Particles are first aligned along the sidewall by the buffer flow. Next, the particles larger than the trapping threshold are captured by the membrane, while other particles are guided through the bypass channel and captured by the subsequent trappers. Once all trappers are occupied, briefly turning the pneumatic off and on releases each trapped particle in sequence. Furthermore, Fallahi et al. proposed a completely stretchable trapping device [306], Figure 5H. The device is entirely fabricated by thin PDMS films and incorporates multiple U‐shaped trapping structures, each with a 10 µm gap. Laterally stretching the whole device can control the gap size of all U‐shaped trappers, thus achieving on‐demand trapping and release of particles and cells.

#### 4.2.4 Particle/Cell Mechanical Deformation

Particle mechanical deformation refers to changing the morphological or mechanical states of particles (such as cells, vesicles, or synthetic microparticles) through hydrodynamic or mechanical forces [318]. In particular, cell deformability may be directly related to the cellular growth status and physiological condition [319, 320]. Leveraging the interactions between cells (or particles) and the channel wall is the most straightforward deformation method, such as cell constriction deformability cytometry [321]. The implementation of soft materials and deformable devices allows adjustable deformation levels for particles and cells.

**FIGURE 6:**
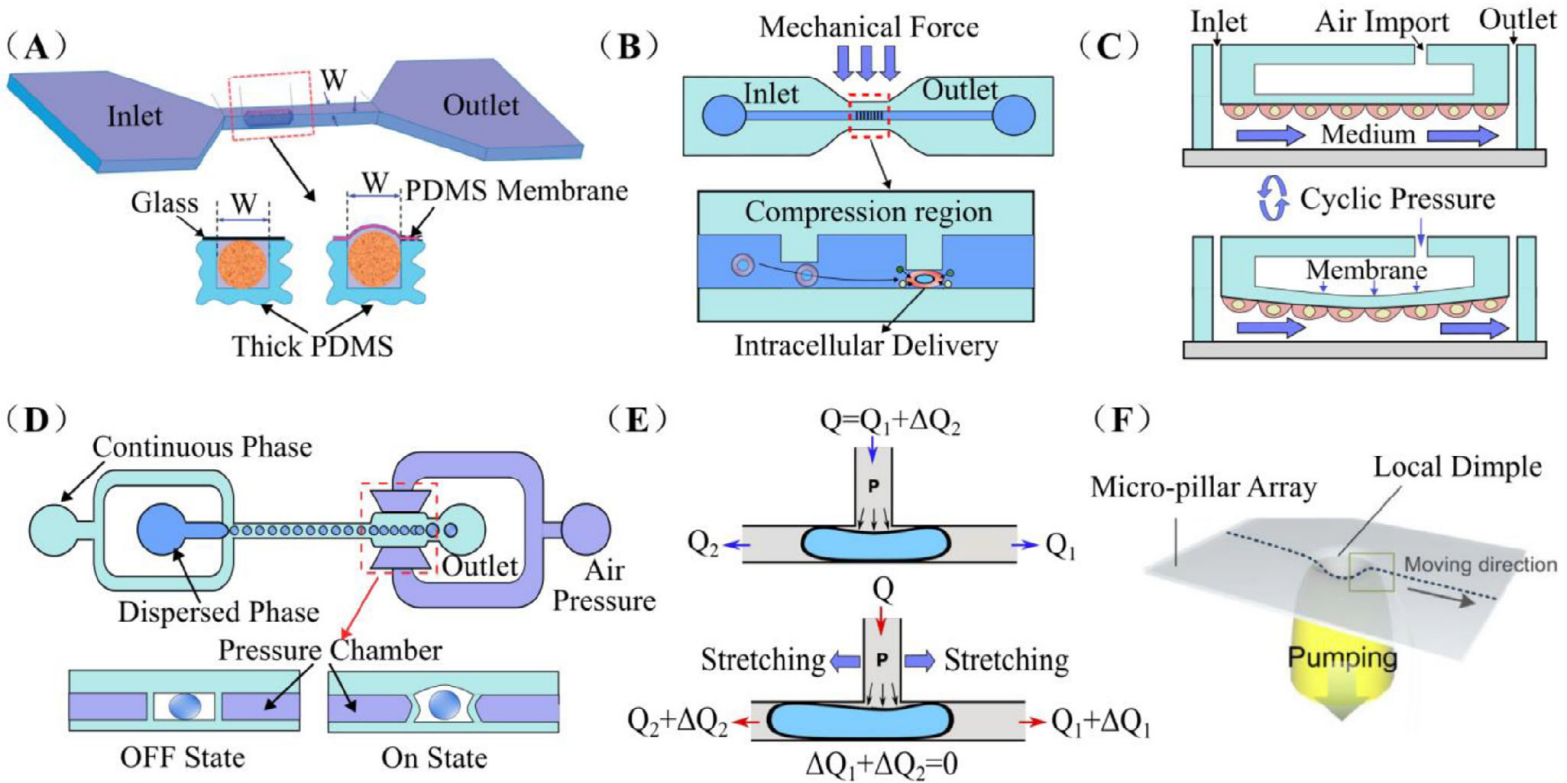
Exploiting device deformability for particle/cell mechanical deformation, droplet manipulation. (A) Cell passage through a constricted compliant channel. Reproduced with permission [307]. Copyright 2018, Royal Society of Chemistry. (B) Size‐tunable cell constriction device. Reproduced with permission [46]. Copyright 2023, Royal Society of Chemistry. (C) Cyclic cell stretching device. Reproduced with permission [42]. Copyright 2023, Elsevier. (D) On‐demand droplet merging device. Reproduced with permission [43]. Copyright 2025, Springer Nature. (E) Tunable droplet splitting device. Reproduced with permission [308]. Copyright 2025, Royal Society of Chemistry. (F) Path‐programmable droplet motion manipulation device. Reproduced with permission [309]. Copyright 2015, Springer Nature.

Squeezing cells through a constriction channel causes temporary deformation by mechanical force, useful in intracellular delivery [322] and cell mechanophenotyping [321]. Cell squeezing device with soft materials can mitigate channel clogging, tune channel size, and alter cell deformation degree [46]. Raj and Sen [307] studied the passage behavior of cells through a partially deformable device, where the top wall of the channel is a thin flexible PDMS membrane, Figure 6A. Compared to a completely rigid channel, the improved compliance of the top soft wall reduces the degree of cell deformation during passage. In addition, external actuation on the flexible membrane can precisely control the constriction size in real‐time, so that the migration and squeezing of cells through the constriction can be controlled, Figure 6B. In this device, a micrometer linear actuator is employed to compress the constriction area on‐demand [46]. Tuning the constriction size can adjust the degree of cell squeezing with different sizes. Moreover, the channel clogging issue can be easily addressed by releasing the pressure.

In addition, mechanically stretching cells adhered to a substrate can mimic cyclic mechanical loading on cells in organisms [323], which is crucial in organ‐on‐a‐chip [324, 325]. Cells are commonly deformed by cyclically stretching or bending the flexible membrane to which they adhere. Figure 6C illustrates a cyclic cell deformation device fabricated by stacking a flexible air chamber, a fluid channel, and a glass substrate [42]. Cells are fixed on the bottom membrane of the air chamber, and cyclic stretching is achieved by applying periodic air pressure to the chamber to deform the membrane.

#### 4.2.5 Droplet Manipulation

Droplets and dispersed liquid beads can be considered as particles in this review after they are segregated from continuous flows. Their kinematic and dynamic behavior could be similarly controlled as the conventional solid particles and cells [326]. Besides, there are additional manipulation methods on droplets such as splitting and merging [327]. Droplet merging aims to combine two or more droplets, thereby allowing reagent addition for multi‐step reactions [328]. Dynamically deforming the merging region can enable on‐demand droplet merging [43], Figure 6D. In this device, two pressure chambers are symmetrically placed on both sides of the droplet merging channel. When pneumatic pressure is applied, the channel narrows in width and expands in height, which reduces the spacing between droplets and induces droplet merging. Different ratios of merged droplets can be induced by varying the pressure levels. Moreover, applying instantaneous pressure can trigger a single controlled merging event.

Droplet splitting refers to dividing a single droplet into two or more small daughter droplets [329]. A T‐junction channel can split droplets where the droplet collides with the wall and is divided into two daughter droplets [326]. Stretching a T‐junction channel changes its dimensions and alters the hydraulic resistance ratio between its two branches, consequently tuning the splitting volume ratios of daughter droplets, Figure 6E [308]. Without stretching, symmetric splitting is achieved, whereas the volume ratio increases to 4 with a stretching strain of 16%.

Furthermore, manipulating the motion and trajectory of a single droplet serves as the basis for merging, mixing, and analysis. The deformability of the micropillar array substrate has been used to manipulate the droplet motion [330]. Figure 6F illustrates a path‐programmable droplet motion method using a thin and flexible superhydrophobic PDMS substrate patterned with micropillar arrays [309]. When a local vacuum is applied below the substrate, a local dimple structure is formed, and the water adhesion force of the structure is reduced. The sloped dimple structure and weakened water adhesive force can precisely enable the motion of individual droplets. In this device, droplets of interest can be captured and released, driven along programmable trajectories, and subjected to the multi‐step merging. Similarly, deformable hydrophobic artificial cilia arrays made of magnetic materials have also been used to manipulate the droplet motion, where local deformation is achieved by a magnetic field [331, 332].

## 5 Applications of Deformable Devices

The flexible materials and their fabrication methods enable the microstructures of devices to undergo on‐demand shape deformation under external actuation, which brings superior advantages in device functionality and adaptability. The deformability of devices can provide tunable and reversible control of microenvironments and enable functions that are difficult in rigid devices. These unique advantages bring up many opportunities for various biomedical and industrial applications. This chapter will highlight the typical applications using engineered device deformability, including the isolation of circulating tumor cells [15, 44], cell mechanophenotyping [45, 333], intracellular delivery [46, 336], drug screening [47, 337], organ‐on‐a‐chip [48, 57, 335], flexible sensors [12, 49, 59, 216], soft robotics [50, 60, 338], and implantable medical devices [13, 339].

### 5.1 Isolation of Circulating Tumor Cells

Cancer liquid biopsy is a minimally invasive diagnostic test that analyzes cancer‐related biomarkers from a simple blood sample (or other body fluids) instead of the traditional tissue biopsy. Isolation of CTCs, the intact cancer cells shed from primary or metastatic tumors into the bloodstream, is of great importance for cancer diagnosis and prognosis [340]. Fallahi et al. have reported a stretchable inertial microfluidic device for the separation of T47D cancer cells from the diluted whole blood [15]. Cancer cells are generally larger than normal blood cells, but they exhibit a broad size distribution. Stretching the flexible device and modifying the channel dimensions can adjust the separation cut‐off size, thus optimizing the separation efficiency.

**FIGURE 7:**
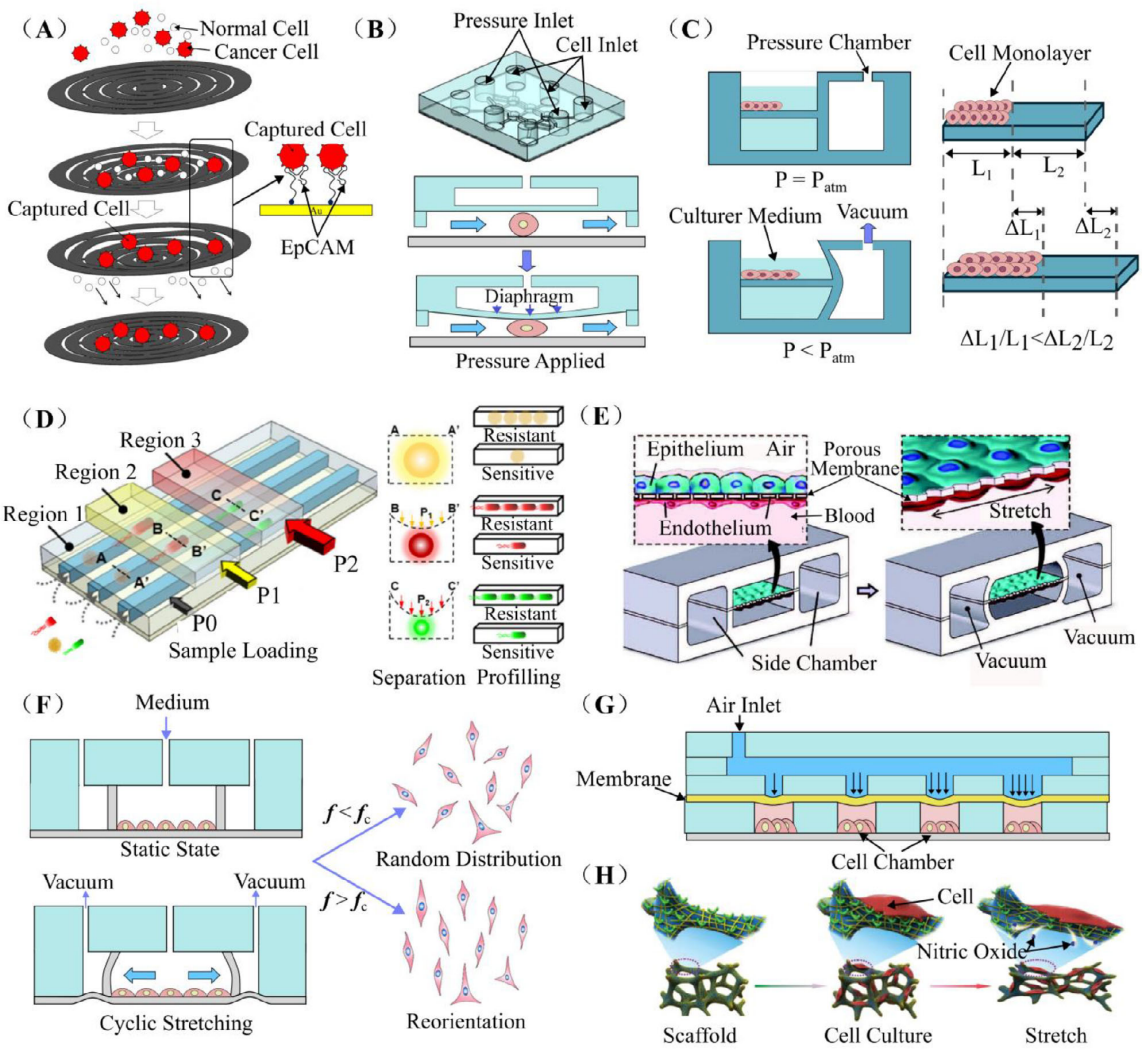
Applications of engineered deformable microdevices in cell isolation, cell mechanophenotyping, drug screening, and organ‐on‐a‐chip. (A) Surface‐modified soft device for the isolation of CTCs. Reproduced with permission [44]. Copyright 2020, AIP Publishing. (B) Cell compression device to measure cell Young's modulus. Reproduced with permission [333]. Copyright 2017, Elsevier. (C) Cell monolayer Young's modulus measurement device. Reproduced with permission [45]. Copyright 2019, Royal Society of Chemistry. (D) Adaptable pathogen classification and antimicrobial susceptibility testing device. Reproduced with permission [47]. Copyright 2019, National Academy of Sciences. (E) Lung‐on‐a‐chip device. Reproduced with permission [57]. Copyright 2010, The American Association for the Advancement of Science. (F) Cyclic stretching enables the cell reorientation device. Reproduced with permission [48]. Copyright 2021, American Chemical Society. (G) Human mesenchymal stem cells proliferation device. Reproduced with permission [334]. Copyright 2007, Royal Society of Chemistry. (H) 3D stretchable and biomimetic cell culture scaffold. Reproduced with permission [335]. Copyright 2021, John Wiley and Sons. Epithelial cell adhesion molecule (EpCAM).

However, the above method is size‐based and is ineffective for separating cells with similar sizes. Kumamoto et al. [341] reported specific binding interactions between the flexible filter and cells, Figure 7A. The filter features a thin gold‐plated nickel disk with multiple slits, and its surface is functionalized with an EpCAM aptamer, which is highly expressed on cancer cells. When a mixture cells sample is injected, the slit expands due to flow pressure, allowing fluid and other cells to flow through, while cancer cells are captured on the surface through the antigen–antibody reactions. This method has been successfully applied for the separation of human breast cancer cells and human embryonic kidney cells [44] as well as the isolation of circulating tumor cells from the blood [342].

### 5.2 Cell Mechanophenotyping

Cell mechanophenotyping is the study and characterization of cellular mechanical properties to understand their physical state, behavior, and biological functions [343]. Mechanical properties are intrinsic markers of cells, including deformability and viscosity, etc. Young's modulus reflects resistance to deformation and serves as a key indicator for disease diagnosis and progression [344]. By deforming the cells in a microchannel via a soft membrane, the Young's modulus of the cells can be directly evaluated [333], Figure 7B. The device includes fluidic channels, pressure channels, cell culture chambers, and diaphragms. Pneumatic pressure deforms the diaphragm, thereby compressing the cell in the culture chamber to varying degrees. The theoretical calculation is based on the assumption that cells are compressed into a flat, symmetrical shape [345]. Young's modulus is then quantified by fitting the cell strain to the applied pressure. This approach has been employed to measure Young's modulus of *Escherichia coli* [333].

In addition, Figure 7C illustrates the method for evaluating the Young's modulus of a cell monolayer via membrane stretching [45]. When a vacuum is applied to the right cavity, the soft wall of the chamber shrinks and consequently stretches the membrane on the left, where half of the membrane area is cultured with a monolayer of cells. This system can be considered as two springs connected in series, one representing the bare membrane and the other representing the composite of cells on the membrane. Therefore, Young's modulus of the cell monolayer can be calculated by subtracting the effect of the bare membrane from the composite.

### 5.3 Intracellular Delivery

Intracellular delivery refers to delivering exogenous cargo into living cells, which can be used for gene therapy, gene editing, and drug delivery [346]. Cell squeezing induces transient membrane perturbation, enabling the passive diffusion of cargo from outside to the interior of the cell [347]. The size‐tunable cell squeezing device shown in Figure 6B has been employed for controlled intracellular delivery [46]. Delivery is modulated by compressing the channel to different degrees through a linear actuator. The transfection efficiency of DNA to HEK293 FT cells increases from 0% to 46% as the constriction dimension decreases from 100 to 5 µm. In addition, pneumatic compression of a membrane has also been used for controlled intracellular delivery of cytoplasmic proteins into (*E. coli)* cells [336].

### 5.4 Drug Screening

Drug screening is the process of testing and evaluating the effects of drugs on biological systems [348]. Microfluidic‐based drug screening has the advantages of low sample consumption and cost‐effectiveness [349]. Soft devices can enhance drug screening efficiency and increase physiological relevance. Deforming the microchannel to form several tunable step filters allows simultaneous classification and antimicrobial susceptibility testing of multiple pathogens [47], Figure 7D. Multiple‐step filters with decreasing gaps are formed by applying pneumatic pressures to the soft channel, which positions bacteria according to their size. When antibiotics are injected, the sensitive or resistant behavior of bacteria can be observed at different positions. In addition, cyclic compression of a cell‐containing channel can enhance the drug screening efficiency [261], Figure 4C. The repeated reciprocating flow generated by compression increases the exposure time of cells to the drug. As a result, the time required for drug‐induced anticoagulation can be reduced severalfold.

Furthermore, organ‐on‐a‐chip devices can mimic the motions of human organs through dynamically structural deformation to recapitulate complex, organ‐level disease processes in humans. These devices become a promising candidate for drug screening [337]. The lung‐on‐a‐chip device shown in Figure 7E has been applied for pulmonary Edema drug development [350]. Pulmonary Edema is first introduced by perfusing interleukin‐2 through the microvascular channel. Then, angiopoietin‐1 and vanilloid 4 are introduced to explore the new therapeutic approaches. Moreover, similar methods have also been demonstrated to screen the drug for radiation‐induced lung injury [351].

### 5.5 Organ‐on‐a‐Chip

Organ‐on‐a‐chip devices replicate the structure, microenvironment, and physiological functions of human organs within a microdevice [337]. These devices have been widely used in drug screening and disease monitoring [352]. Deformable microchannels, microchambers, and other microstructures are employed to culture organ‐specific cells while undergoing mechanical deformation, thereby simulating stimuli and motions in physiological environments. Huh et al. [57] developed a pioneering lung‐on‐a‐chip device that mimics the lung's blood‐air barrier by integrating dynamic perfusion with cyclic stretching of a porous membrane, Figure 7E. The device comprises two pneumatic chambers with thin walls, an air channel, a liquid channel, and a porous membrane separating the channels. Alveolar epithelial cells are cultured on the air‐facing side of the membrane, while microvascular endothelial cells are seeded on the opposite side, exposed to liquid flow to simulate blood‐cell substances exchange. The porous membrane enhances the interactions between the two cell types, while cyclic vacuum applied to the side chambers stretches the membrane and simulates the mechanical deformation of lung cells during breathing. In addition, the stretchability of the soft membrane can be employed to investigate the cell reorientation. Cells with initially random orientations rotate and align to a well‐defined angle in response to cyclic strain and shear force [48], Figure 7F. In this system, human mesenchymal stem cells are cultured on a surface‐modified membrane through which culture medium flows. Cyclic vacuum is applied to the air chamber to stretch the membrane and cells simultaneously. As a result, cells with an initial random orientation are reoriented perpendicular to the stretching direction when the applied frequency exceeds a certain threshold.

Cyclic compression of soft culture chambers can mimic the proliferation process of human mesenchymal stem cells (hMSCs) [334], Figure 7G. A pneumatic inlet is branched into five channels with different diameters to apply compressive stimuli with various amplitudes to the microchambers. A moderate level of cyclic stimuli can enhance the proliferation of hMSCs, while excessive stimulation hinders cell proliferation. More recently, Qin et al. developed a 3D flexible and biomimetic scaffold for a bone‐on‐a‐chip device [335], Figure 7H. The scaffold is fabricated by attaching the networks of peptide‐linked gold nanotubes onto porous PDMS, enabling it to stretch, bend, and twist. Chondrocytes are cultured on the surface of the scaffold, where the nanotubes detect biochemical signals generated by cells under varying deformations to explore the mechanism of osteoarthritis.

### 5.6 Flexible Sensors

**FIGURE 8:**
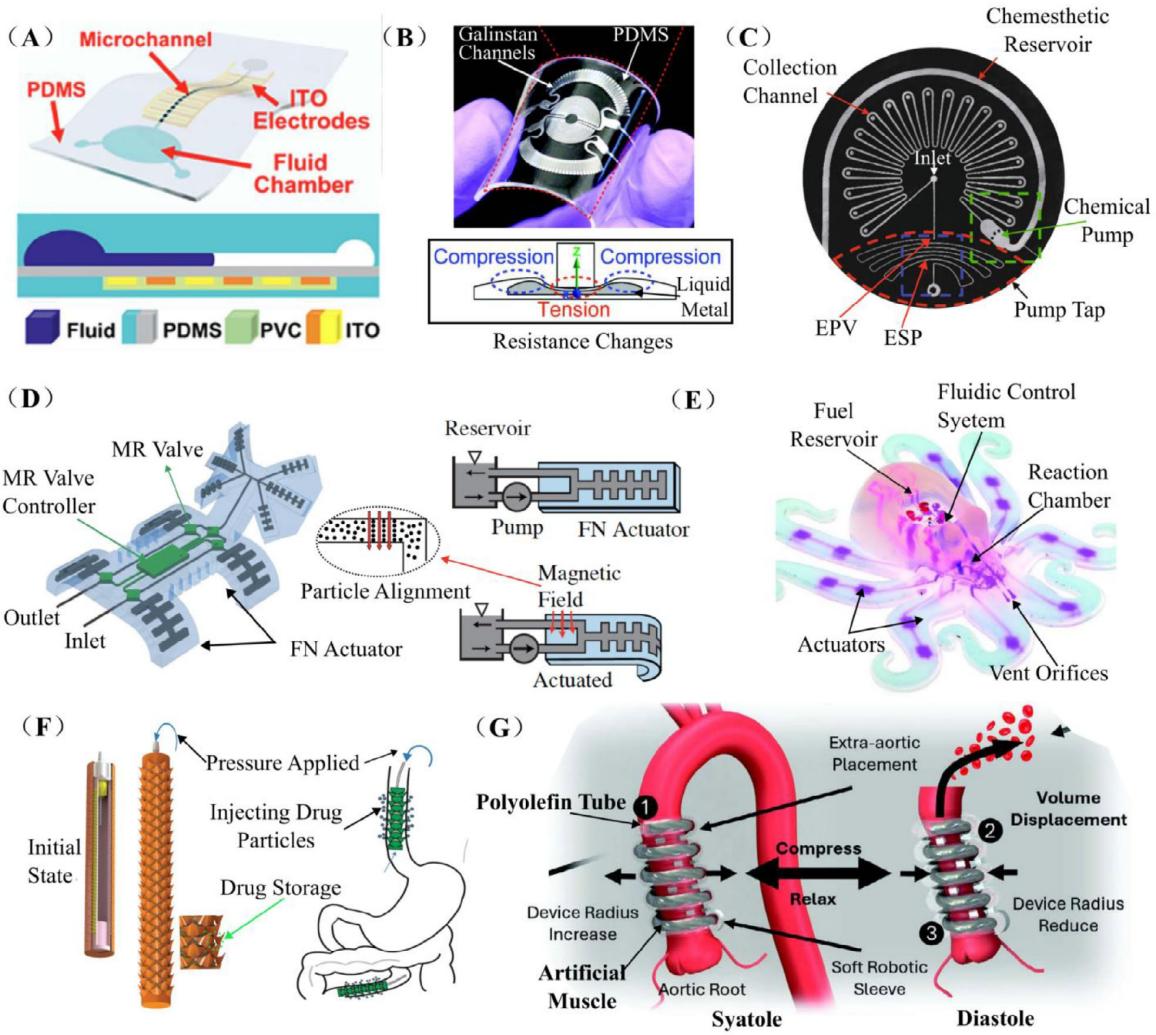
Applications of deformable microdevices in flexible sensors, soft robotics, and implantable medical devices. (A) Flexible microfluidic triboelectric sensor. Reproduced with permission [59]. Copyright 2023, Elsevier. (B) Metal–liquid‐based flexible pressure sensor. Reproduced with permission [49]. Copyright 2017, John Wiley and Sons. (C) Hand‐operated flexible sweat sensor. Reproduced with permission [216]. Copyright 2019, Springer Nature. (D) Magnetorheological fluid‐based soft robot. Reproduced with permission [50]. Copyright 2020, John Wiley and Sons. (E) Self‐powered soft robot. Reproduced with permission [338]. Copyright 2016, Springer Nature. (F) Soft hollow cylindrical stents for local drug delivery. Reproduced with permission [13]. Copyright 2021, Springer Nature. (G) Liquid‐driven hemodynamic stabilization device. Reproduced with permission [339]. Copyright 2024, John Wiley and Sons. Fluid‐net (FN).

Flexible mechanical sensors detect mechanical stimuli such as pressure, strain, shear force, and bending, playing a crucial role in soft robotics, wearable electronics, and artificial intelligence [58]. Microfluidic‐based flexible mechanical sensors integrate microchannels filled with liquid into soft substrates, detecting the force through electrical or chemical responses induced by channel deformation or fluid redistribution [62]. Figure 8A illustrates a flexible microfluidic triboelectric sensor designed for pressure and bending angle measurements [59]. When pressure is applied, liquid is pumped from the chamber and flows along the channel. Once the liquid flows over an ITO electrode, a voltage peak is generated due to the triboelectric effects. Since a higher applied force produces a greater number of peaks, the applied pressure or bending angles can be measured by counting the voltage peaks. In addition, liquid metal is a promising material in flexible sensors due to its electrical conductivity and fluid properties [147]. LM is generally embedded in a microchannel within the thin membrane. Stretching or pressing the flexible membrane changes the cross‐section or the length of the LM channels, thus changing their electrical resistance [149]. Generally, the LM channel is connected as a Wheatstone bridge to convert resistance changes into amplified voltage signals for pressure and strain detection [353]. The pressure sensor shown in Figure 8B has been employed for heart rate monitoring, where each heartbeat compresses the sensor and generates a voltage signal in a short time [49].

Flexible sweat sensors allow continuous, noninvasive collection and analysis of sweat, which provides insight into human physiology and health status [354, 355]. A large number of sweat sensors depend on passive forces such as capillary force to collect sweat, lacking controllability and precision [356]. In contrast, deformable valves or pumps have also been integrated within the sensors to achieve adjustable and accurate sweat flow control. Roger's group developed a deformable, hand‐operated, resettable sweat collecting and warning sensor [216], Figure 8C. The sensor consists of an elastomeric pinch valve (EPV), an elastomeric suction pump (ESP), a collection chamber, and a chemesthetic reservoir. Sweat is collected via the inlet in the center. Stretching the pump tap can open the EPV and generate negative pressure through ESP, expelling the collected sweat and resetting the system. When the collection reaches its maximum volume, the foaming agent in the reservoir is activated, delivering the chemical stimulant to the skin to alert the user. In addition, temperature‐sensitive hydrogel has been employed to fabricate a programmable sweat analysis sensor [12]. The sensor has a central sweat inlet surrounded by multiple reaction chambers for different tests, each equipped with a temperature‐sensitive hydrogel microvalve. A programmable circuit is connected and can selectively heat these microvalves, allowing precise control and analysis of sweat.

### 5.7 Soft Robotics

Soft robotics are a class of robotic systems entirely made of soft materials, achieving motions by continuously deforming, bending, elongating, or twisting their flexible actuators [357]. Compared to traditional rigid robots, soft robots are compliant, adaptable, and easy to achieve dexterous movement [358]. Whitesides's group first introduced the concept of soft robotics and developed a multigait soft robot [60]. The robot includes five FN actuators, each composed of multiple air chambers embedded in a flexible elastomer and bonded to an inextensible layer. When pneumatic pressure is applied, the FN actuators bend due to the strain difference between the elastomeric layer and the inextensible bottom. Crawling, undulation gaits, and obstacle avoidance behaviors can be achieved by programming the five air pumps. Moreover, a magnetorheological (MR) fluid‐based robot has been developed based on the FN actuators [50], Figure 8D. MR fluid channels and valves are integrated within a robot with five FN actuators. When a magnetic field is applied to a specific valve, the magnetic particles in the MR channel redistribute, directing the fluid through the FN actuators and inducing bending. Versatile robotic motions can be achieved by configuring magnetic fields on demand. However, these robots need external pneumatic or hydraulic actuation, restricting their autonomy and mobility. More recently, an entirely self‐powered family of soft robots is emerging [338]. One example is shown in Figure 8E. This robot burns fuel internally to generate gas, which is then delivered to a fluidic system to drive and control its motion in a prescribed pattern. Whitesides provided a more comprehensive review of soft robotics [63].

### 5.8 Implantable Medical Devices

Soft materials play an important role in implantable medical devices, as their flexibility allows them to function in the narrow, complex, or tortuous human organs [359]. Implantable drug delivery systems precisely transport drugs to target organs, fulfilling therapeutic demands while minimizing systemic side effects. Figure 8F shows a pneumatic soft hollow cylinder for delivering drugs to a tubular organ such as the gastrointestinal tract [13]. The device features a hollow cylindrical actuator surrounded by a periodic array of snake‐denticle‐shaped kirigami skin. Once the device reaches the target organs, pneumatic pressure is applied to the hollow cylinder, causing it to expand. This expansion forces the kirigami needles to buckle outward and contact the organ, thereby releasing the drugs.

Hemodynamic stabilization is crucial in managing acute cardiac events, and it can be enhanced by periodic aortic compression using a flexible tube [339], Figure 8G. This device embeds stretchable hydraulic artificial muscles into a customized helical polyolefin tube. The assembled device is implanted and wrapped around the ascending aorta, operating in synchrony with the cardiac cycle. During systole, the hydraulic pressure is applied to the artificial muscles before the aortic valve opens, causing them to elongate. Consequently, the polyolefin tube detaches from the aorta, lowering aortic root pressure and reducing left ventricular afterload. In contrast, the device compresses the aorta during diastole, thereby increasing aortic pressure and enhancing end‐organ perfusion. This device has shown potential as a medium‐term extra‐aortic counterpulsation therapy.

## 6 Discussion and Outlooks

In this paper, we first summarized the main materials in deformable devices, including soft polymers and other supplementary materials. Next, we discussed the prototyping and bonding techniques for the fabrication of deformable devices. Subsequently, we elaborated the deformability‐enabled functionalities in fluid (e.g., valving, pumping, and mixing) and particle manipulation (e.g., focusing, sorting, as well as trapping and release). We explained how partial and full deformation of devices can enable or enhance these functionalities. Finally, we reviewed the main applications of deformable microdevices in biomedicine and industry, such as isolation of CTC, cell mechanophenotyping, intracellular delivery, flexible sensors, soft robotics, and implanted medical devices. We developed a table to indicate the relationship between materials, deformation formats, property rationales for materials selection and potential alternative materials, in Table 2 to facilitate readers in designing their devices. Driven by advancements in soft matter and manufacturing techniques, this field has undergone rapid development over the last few decades. Despite impressive demonstrations, widespread adoption is inhibited by interlinked challenges in device materials, fabrication methods, and actuation mechanisms.

**TABLE 2:** Materials selection and implementation mapping table.

Materials	Functional category	Reference	Deformation format	Property rationale for material selection	Potential alternative materials
PDMS	Valving	Figure 3B Refs. [34, 257, 268]	The PDMS thin membrane is bent to produce global or local obstacles, thereby changing the fluid flow state or particle dynamics.	• Elastic deformability of PDMS membrane or whole device • Easy molding • Easy plasma bonding • Fatigue resistance • Optical transparency • Biocompatibility	PDMS‐Ecoflex /Dragon Skin hybrids
	Pumping	Figure 3D,E Refs. [35, 54, 256, 284, 285]			
	Fluid regulator	Figure 4A,B Refs. [53, 258, 293]			
	Mixing	Ref. [35]			
	Droplet generation	Figure 4F Refs. [17, 263]			
	Particle sorting	Figure 5B Refs. [40, 47]			
	Particle trapping and release	Figure 5F,G Refs. [41, 306]			
	Particle/Cell mechanical deformation	Figure 6A,B Refs. [46, 307, 333]			
	Droplet merging	Figure 6D Ref. [43]			
	Flexible sensors	Figure 8A,B Refs. [49, 59, 216]			
	Particle/Cell mechanical deformation	Figure 6C Refs. [42, 45]	The PDMS thin membrane is cyclically bent or stretched to periodically deform the cell.		
	Organ on a chip	Figure 7E–G Refs. [48, 57, 334]			
	Valving	Figure 3C Ref. [209]	The whole PDMS device is stretched to modify the geometry and size of the channel or microstructure, thus changing the fluid flow state or particle dynamics.		
	Mixing	Figure 4D Ref. [55]			
	Droplet generation	Figure 4G Ref. [38]			
	Particle focusing	Figure 5A Ref. [15]			
	Particle sorting	Figure 5D Ref. [56]			
	Particle trapping and release	Figure 5H Ref. [306]			
	Droplet splitting	Figure 6E Ref. [308]			
	Pumping	Figure 3F Refs. [151, 260, 287]	PDMS micropillars embedded with magnetic particles are actuated by a magnetic field to swing within the channel, thereby generating net flow, introducing vortices in the fluid, or guiding the specific motion of particles.	• Elastic deformability of PDMS micropillars • Low viscosity to fill the module	
	Mixing	Figure 4E Ref. [37]			
	Particle motion	Ref. [76]			
	Droplet motion	Refs. [331, 332]			
Ecoflex	Fluid oscillator	Figure 4C Ref. [261]	An Ecoflex chamber is cyclically deformed to periodically compress and release the fluid channel to introduce oscillatory flow.	• Elastic deformability • Easy molding • Large elongation at break • Good optical transparency • Fatigue resistance • Biocompatibility	PDMS‐Ecoflex /Dragon Skin hybrids, or Dragon Skin
	Particle focusing	Ref. [39]	The thin Ecoflex device with a straight channel is stretched to over 300% to increase the length of particle migration.		
	Particle sorting	Refs. [18, 82]			
	Soft robotics	Figure 8D,E Refs. [50, 60, 338]	The Ecoflex fluid channel layer is bonded to a strain‐limiting layer to induce the controlled bending required for robot motion.		
Dragon Skin	Flexible sensors	Ref. [149]	Dragon Skin membrane serves as the sensor substrate and isolates multiple layers of liquid metal.	• Elastic deformability • High tear strength • Electrical insulation • Biocompatibility • Similar mechanical properties to human aorta	PDMS‐Ecoflex /Dragon Skin hybrids
	Soft robotics	Figure 8D Ref. [50]	Dragon Skin is used to fabricate soft robotic joints that enable bending.		
	Implantable medical devices	Figure 8G Ref. [339]	Dragon Skin is used as a material for an artificial human aorta.		
PDMS‐Ecoflex /Dragon Skin hybrids	Flexible sensor	Ref. [87]	PDMS‐Ecoflex hybrids membranes are used to seal liquid metal to construct a strain sensor.	• Elastic deformability • Easy Plasma bonding • Easy molding • Electrical insulation	—
	Soft robotics	Ref. [92]	PDMS‐Dragon Skin hybrids are used to fabricate soft robotic joints that enable bending.		—
Parylene C	Pressure sensor	Ref. [109]	Parylene C membrane is used to seal the capacitive chamber and sense pressure.	• Elastic deformability	Flexdym
PMMA	Valving	Figure 3A Ref. [51]	PMMA is used as a substrate to withstand pressure and maintain the structural and dimensional stability of devices.	• High mechanical rigidity • Optical transparency • Thermal bonding capability	Glass
	Pumping	Ref. [284]			
Flexdym	Particle sorting	Figure 5E Ref. [14]	The thin Flexdym device with a straight channel is stretched laterally to adjust the aspect ratio in a wide range to enable tunable particle sorting.	• Large elongation at break • Thermal bonding capability • Optical transparency • Biocompatibility	TPU
TPU	Valving	Figure 3A Ref. [51]	The TPU thin membrane is bent to control the opening and closing of fluid ducts.	• Elastic deformability • Thermal bonding capability	Flexdym
Polyimide	Flexible sensor	Ref. [360]	Polyimide membrane serves as a dielectric layer in flexible tactile sensors.	• Elastic deformability • Electrical insulation	PDMS, Ecoflex, Dragon Kin
Stimuli‐sensitive hydrogel	Particle sorting	Figure 5C Refs. [19, 133]	Micropillars made of stimulus‐responsive hydrogels can swell or shrink when stimulated (pH, temperature) in fluid flow, thus adjusting the gaps in the micropillar array and achieving tunable sorting.	• Stimuli‐responsiveness • Swell and shrink capability	—
Glass	Pumping	Figure 3E(ii), Ref. [35]	Glass is used as a substrate to withstand pressure and maintain the structural and dimensional stability of devices.	• High mechanical rigidity • Optical transparency. • Easy plasma bonding • Chemical inertness	—
	Droplet generation	Figure 4F Ref. [263]			
	Particle sorting	Figure 5B Ref. [40]			
	Particle/Cell mechanical deformation	Figure 6C Ref. [42]			

The ideal flexible materials should have high stretchability, ease of fabrication and bonding, suitable mechanical strength, and good biocompatibility. PDMS is the most widely used material in deformable devices with excellent mechanical strength. It can be easily bonded to itself or other silicon‐based materials through plasma treatment. However, the limited stretchability of PDMS (maximum strain of 120% at a base‐to‐curing agent ratio of 10:1 [67]) limits its use in applications requiring large strain. In contrast, other commercial silicon elastomers such as Ecoflex and Dragon Skin have a superior stretchability with the maximum strain of 700%–900% [68], but these elastomers cannot be easily bonded through plasma treatment. Embedding a soluble sacrificial module (e.g. metal wire) within an Ecoflex substrate can fabricate microchannels in an ultrastretchable device [39, 82]. However, this approach is limited to devices with simple geometries. Recently, PDMS‐Ecoflex/ Dragon Skin hybrids at a specific ratio exhibit excellent stretchability (e.g., PDMS‐Dragon Skin 1:3 with a maximum strain of 230% [92]) and are capable of plasma bonding, demonstrating its good potential in deformable devices. The systematic study on the mechanical strength, plasma bonding performance, and biocompatibility of these hybrid materials is still needed to evaluate their feasibility, and the practical applications of the materials are also largely under explored.

Meanwhile, new fabrication and processing methods are in demand to enhance the structural accuracy and reliability of deformable devices. The current high‐resolution microfabrication techniques are inherently designed for rigid materials, such as photolithography, etching, and micromilling. Application of these techniques for machining soft materials suffers from poor dimensional accuracy since the soft materials may shrink, expand, or bend during the process. The most common fabrication for deformable devices relies on soft lithography, which offers high resolution but is limited in constructing 2D layered geometries. Most 3D geometries can only be achieved by stacking multiple layers through plasma bonding [179]. This time‐consuming and cumbersome process significantly raises the risk of fluid leakage and device failure for long‐term use. Moreover, soft layers could also stretch or warp, which may cause misalignment in multilayer structures. The sacrificial mold‐assisted replication is promising for fabricating geometrically complex structures without bonding [361], but the effective removal of the sacrificial materials is a challenge, especially for complex 3D geometries and small dimensions. Additive manufacturing or 3D printing enables the direct fabrication of complex structures in devices in a single step. For example, two‐photon polymerization (TPP) is an emerging 3D printing technique that utilizes two photons to induce localized polymerization of materials [362]. This technique can fabricate complex and compliant hydraulic actuators in submicron resolution [363]. However, the current high cost of TPP equipment and the slow fabrication speed hinder its applications for printing large components. Developing high‐precision, high‐efficiency, and low‐cost 3D printing techniques and tailoring the techniques for soft materials is still in demand in this field.

Roll‐to‐roll (R2R) sheet processing is a continuous manufacturing method in which a long, flexible substrate—such as plastic film, metal foil, or paper—is unwound from a supply roll, processed through steps including coating, printing, drying, or laminating, and then rewound onto a take‐up roll [364]. This approach enables high‐speed, high‐volume, and cost‐efficient production of products such as flexible electronics, solar cells, packaging films, and labels. R2R sheet processing offers continuous, low‐cost manufacturing with efficient material utilization and consistent quality [365, 366]. Integrating TPP with R2R sheet processing may provide submicron to nanoscale precision from TPP, combined with the high throughput and cost benefits of R2R, for the fabrication of soft, layered devices. This integration allows patterning precision microstructures in localized regions (e.g., at junctions, gates, or valves) using TPP, while the bulk layers are produced rapidly via R2R coating or lamination. The effectiveness of this combined approach depends on the synchronization of TPP printing and R2R lamination, material compatibility, and strong adhesion between multilayers.

Furthermore, novel actuation mechanisms are needed to enable more robust device functionality and a self‐contained power supply and control. To date, mechanical, pneumatic, and hydraulic actuations are the most common methods. However, these actuations require bulky external equipment for mechanical movement and pressure generation, which limits their portability and autonomy. Developing smart actuation mechanisms in a distant and contactless way is important for certain applications where devices need to function in narrow and confined spaces, such as implantable medical devices, minimally invasive surgery tools, and pipeline inspection robots, etc. MR materials, comprising magnetic particles in elastomer matrices, can be activated and controlled in a noncontact manner. MR membranes, thin channels, and pillars can be controlled by a distant magnetic field to control fluid pumping, mixing, and soft robots [35, 37, 50]. Moreover, entirely self‐powered and controlled actuation is another alternative solution. For example, a robot developed by Wehner et al. [338] generates working gas for the actuation via internal fuel combustion, serving as a pioneer attempt. However, the current one can only perform preprogrammed motions. Therefore, future efforts are needed to improve the versatility and real‐time controllability of the platform.

In conclusion, exploiting the deformability of devices can enhance the performance in both fluid and particle manipulation. These advancements have already been widely applied for disease diagnosis, therapeutic development, organ‐on‐a‐chip, drug screening, and soft robotics, etc. Although significant progress has been made, further efforts are still required in materials, fabrication and processing, and the actuation mechanism. With the continuous development of flexible electronics, wearable devices, and soft robotics, this field is expected to attract growing attention, driving the creation of devices and actuation mechanisms that are more intelligent, flexible, multifunctional, and compliant.

## Author Contributions

J.Z. performed conceptualization, supervision, and project administration. Z.H. performed formal analysis, wrote the original draft, and visualization. X.K. contributed to visualization. D.Y., N.‐T.N., and J.Z. reviewed and edited the draft. N.‐T.N. and J.Z. performed funding acquisition. All the authors provided critical feedback and read and approved the manuscript.

## Conflicts of Interest

The authors declare no conflicts of interest.

## Data Availability

The authors have nothing to report.
